# European mushroom assemblages are darker in cold climates

**DOI:** 10.1038/s41467-019-10767-z

**Published:** 2019-06-28

**Authors:** Franz-Sebastian Krah, Ulf Büntgen, Hanno Schaefer, Jörg Müller, Carrie Andrew, Lynne Boddy, Jeffrey Diez, Simon Egli, Robert Freckleton, Alan C. Gange, Rune Halvorsen, Einar Heegaard, Antje Heideroth, Christoph Heibl, Jacob Heilmann-Clausen, Klaus Høiland, Ritwika Kar, Håvard Kauserud, Paul M. Kirk, Thomas W. Kuyper, Irmgard Krisai-Greilhuber, Jenni Norden, Phillip Papastefanou, Beatrice Senn-Irlet, Claus Bässler

**Affiliations:** 1 Plant Biodiversity Research Group, Department of Ecology & Ecosystem Management, Technische Universität München, 85354 Freising, Germany; 2 Bavarian Forest National Park, 94481 Grafenau, Germany; 3 Department of Geography, University of Cambridge, Cambridge, CB2 3EN UK; 4 Research Unit Biodiversity & Conservation Biology, Swiss Federal Research Institute WSL, 8903 Birmensdorf, Switzerland; 5 Global Change Research Centre and Masaryk University, 61300 Brno, Czech Republic; 6 Field Station Fabrikschleichach, Department of Animal Ecology and Tropical Biology, Biocenter University of Würzburg, 96181 Rauhenebrach, Germany; 7 Norwegian Institute for Nature Research, Gaustadalléen 21, NO-0349 Oslo, Norway; 8 School of Biosciences, Cardiff University, Cardiff, CF10 3AX UK; 9 Department of Botany and Plant Sciences, University of California, Riverside, CA 92521 USA; 10 Department of Animal & Plant Sciences, University of Sheffield, Sheffield, S10 2TN UK; 11 School of Biological Sciences, Royal Holloway, University of London, Egham, Surrey, TW20 0EX UK; 12 Natural History Museum, University of Oslo, Blindern, 0318 Oslo Norway; 13 Norwegian Institute of Bioeconomy Research, 5244 Fana, Norway; 14 Ecology Research Group, Department of Biology, Philipps Uuniversity Marburg, 35043 Marburg, Germany; 15 Center for Macroecology, Evolution and Climate, Natural History Museum of Denmark, University of Copenhagen, 2100 Copenhagen, Denmark; 16 Centre for Plant Molecular Biology, Developmental Genetics, University of Tübingen, 72076 Tuebingen, Germany; 17 Mycology Section, Jodrell Laboratory, Royal Botanic Gardens Kew, Surrey, TW9 3DS UK; 18 Department of Soil Quality, Wageningen University, 6700 AA Wageningen, The Netherlands; 19 Division of Systematic and Evolutionary Botany, Department of Botany and Biodiversity Research, University of Vienna, 1030 Vienna, Austria; 20 TUM School of Life Sciences Weihenstephan, Land Surface–Atmosphere Interactions, Technical University of Munich, 85354 Freising, Germany; 21 Technical University of Munich, Chair for Terrestrial Ecology, 85354 Freising, Germany

**Keywords:** Evolutionary ecology, Macroecology, Fungal ecology, Fungal evolution

## Abstract

Thermal melanism theory states that dark-colored ectotherm organisms are at an advantage at low temperature due to increased warming. This theory is generally supported for ectotherm animals, however, the function of colors in the fungal kingdom is largely unknown. Here, we test whether the color lightness of mushroom assemblages is related to climate using a dataset of 3.2 million observations of 3,054 species across Europe. Consistent with the thermal melanism theory, mushroom assemblages are significantly darker in areas with cold climates. We further show differences in color phenotype between fungal lifestyles and a lifestyle differentiated response to seasonality. These results indicate a more complex ecological role of mushroom colors and suggest functions beyond thermal adaption. Because fungi play a crucial role in terrestrial carbon and nutrient cycles, understanding the links between the thermal environment, functional coloration and species’ geographical distributions will be critical in predicting ecosystem responses to global warming.

## Abstract (web summary)

The functions of color in fungi are not well characterized. Here, Krah and colleagues investigate the color of mushroom assemblages across Europe and show relationships with climate, nutritional mode (saprotrophic and ectomycorrhizal) and seasonality.

## Introduction

**Fig. 1:**
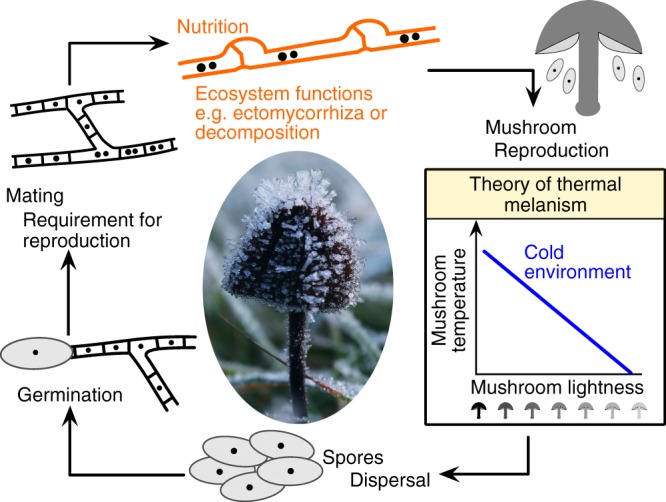
The theory of thermal melanism for multicellular mushroom-forming fungi. The theory predicts that dark-colored mushrooms heat up more rapidly than light-colored mushrooms and, therefore, have advantages, such as increased reproductive success, in cold environments. Mushroom photo taken from http://www.peakpx.com/556831/brown-mushroom-coated-with-snow under Creative Commons (CC0). The mushroom illustration was drawn by Franz-S. Krah using the program Affinity Designer

Ectothermic animals with dark-colored bodies warm up more rapidly than those with light-colored bodies^1–4^, and therefore have a fitness advantage due to increased survival, growth and reproduction in cold environments (“Bogert’s rule”^3^). This theory of “thermal melanism”^1^ has often been studied in the animal kingdom^2,3,5–10^, however, much less is known about the function of colors within the ectothermic fungal kingdom^11–13^. A recent study found that dark pigmentation in yeasts increases heat capture from radiation, which affects fitness depending on ambient temperatures, and affects their latitudinal distribution^14^. Mushrooms are the multicellular reproductive organs of many fungi^15^, with extraordinary diversity in their pigmentation^16^, however the causes and consequences of their coloration are largely unknown^13^. The main pigments of mushrooms are melanins, a diverse group of black to brown polymers, and the more colorful substances such as quinones, arylpyruvic acid derivatives, styrylpyrones, and russupteridins^16^. Mushroom fruit bodies, consisting of a pileus (cap) and stipe (stalk), have the biological function to produce and release sexual spores, which develop into mycelia that exploit resources and mate to form new reproductive individuals^17^. Mycelia are temporally more persistent than fruit bodies, and dark pigmented (e.g., melanized) mycelia are prevalent in cold Arctic and Antarctic environments^18,19^. However, pigmentation may also be an important trait of mushroom fruit bodies in cold environments because they are above-ground reproductive organs and thus are temporarily more exposed to cold temperatures than the mycelium within the substrate (Fig. 1). Thus, we test whether the lightness of mushroom assemblages is correlated with the thermal environment across Europe.

Temperature varies both spatially, across macroclimatic gradients (Supplementary Fig. 1), and temporally. To fully consider this spatiotemporal variability, we examined three measures of temperature: (i) mean temperature (thermal component 1), (ii) variability of temperature (thermal component 2), and (iii) seasonality (month). However, mushroom color might also be affected by other functions of pigmentation than thermal melanism. For example, melanin has been hypothesized to increase pathogen resistance in humid habitats and ultraviolet (UV) protection for ectotherm insects^6,7,10^. Furthermore, other mechanisms of thermoregulation related to habitat characteristics (e.g., microclimate) might likewise be important for fungi^20,21^. These factors could offset the importance of thermal melanism for mushroom-forming fungi and were therefore included in our analyses.

**Fig. 2:**
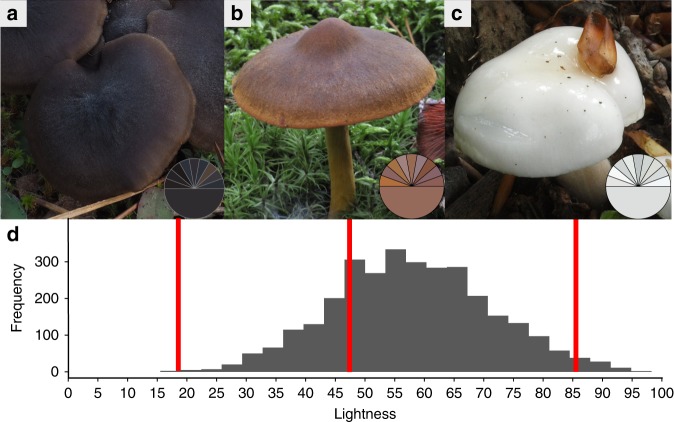
Histogram of mushroom lightness. **a**–**c** Three exemplar species are shown with their respective color lightness (upper half of the pie chart represents each of the 9 single cap measurements; lower half of the pie chart the respective mean). Species from left to right: **a** Entoloma sericeum (color lightness: 18), **b** Cortinarius semisanguineus (color lightness: 47), **c** Hygrophorus eburneus (color lightness: 86). **d** Histogram of color lightness of 3054 mushroom-forming species (color range from 18 to 98). Photos by Peter Karasch (**a**) and Franz-S. Krah (**b**, **c**). Source data underlying **d** are provided in Supplementary Data 1, 2, and 3

We used a Europe-wide dataset of 3.2 million observations of 3054 mushroom-forming fungal species (Supplementary Table 1) from four orders within the systematic class Agaricomycetes (Basidiomycota), spanning eight countries and 40 years^22^. We measured mushroom color on representative digital images of each species by decomposing color into three independent measures: hue, saturation, and lightness (HSL color space). This method precisely differentiated the lightness values of the fungal species based on 29,490 color samples (Supplementary Fig. 2). Scaled from 0 to 100, lightness followed a Gaussian distribution around a mean of 58 (range 18.0–97.8, Fig. 2). We further generated a mega-phylogeny consisting of 2057 mushroom-forming species using publicly available DNA sequence data (Supplementary Fig. 3).

Two predominant nutritional modes (lifestyles) of carbon acquisition are present in mushroom-forming fungi: saprotrophic and symbiotic (ectomycorrhiza, ECM). Fungi from both nutritional modes perform important ecosystem processes. As symbionts, mutualistically associated with plant roots of many tree species^23^, ECM fungi receive carbon from their host plants and in return increase mineral nutrition, water uptake, and resistance against pathogens of their host trees^24,25^; thus, ECM fungi shape the structure and productivity of many forest ecosystems. Saprotrophs, in contrast, are the major decomposers of dead organic matter and release nutrients, which is crucial in carbon and nutrient cycling^26^.

The functional role of mushrooms of both ectomycorrhizal and saprotrophic fungi is the production and release of sexual spores^17^. However, saprotrophs and ECM fungi differ in their fruiting phenology^27,28^ and fruit body-related life history traits^29–31^. Previous studies have shown that ECM fungi invest more in reproductive traits than saprotrophs, which has been attributed to the mutualistic lifestyle^29,32^. For example, ECM fungi produce larger fruit bodies than saprotrophs which, even considering that saprotrophs often produce more fruit bodies, yield a larger biomass^33^. Nevertheless, mean fruit body size and number of fruit bodies of assemblages of both nutritional modes showed a similar response along a productivity gradient^34^. Synthesis of mushroom pigments is costly^35^ and based on the hypothesis that pigmentation increases reproductive fitness, we would expect that ECM species are darker than saprotrophic species. Nevertheless, given the same function of the fruit body in both lifestyles, we would expect saprotrophic and ectomycorrhizal fungi to show similar responses of color lightness along temperature gradients. Based on the lightness of each species, we have therefore calculated the average assemblage-based color lightness (hereafter “mushroom color lightness”) separately for saprotroph and ECM fungi at a European grid resolution of 50 km × 50 km for each month. We tested whether: (1) the lightness of mushrooms differs between saprotrophic and mutualistic ectomycorrhizal species; and (2) whether the response of mushroom color lightness to temperature (spatially and temporally) is consistent with the thermal melanism theory.

Our analyses show that mushrooms of ectomycorrhizal species are darker than saprotrophic species. Using the average assemblage lightness, we show that ectomycorrhizal and saprotrophic mushroom assemblages are darker in cold environments supporting the theory of thermal melanism. An analysis of the seasonality of mushroom color lightness shows that saprotrophic assemblages respond consistent with the theory of thermal melanism—darker in spring and fall, lighter in summer—whereas ectomycorrhizal fungi do not show this pattern. This indicates a more complex ecological role of mushroom colors.

## Results

### Color lightness of mushrooms of the nutritional modes

**Fig. 3:**
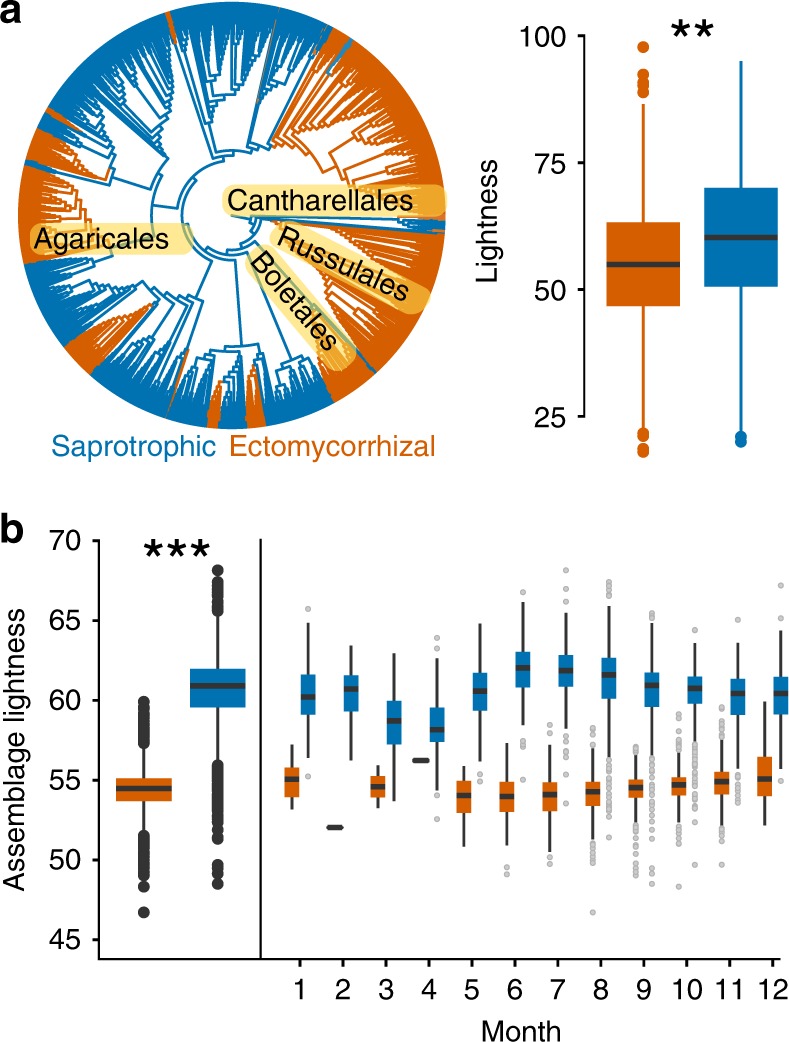
Differences in mushroom color lightness between nutritional modes. **a** Phylogenetic distribution of saprotrophic (blue) and ectomycorrhizal fungal species (orange). Boxplot shows the lightness of the two nutritional modes of fungi (ectomycorrhizal and saprotrophic fungi) and the test results based on phylogenetic linear regression (model Lambda; *z*-value = 4.06; *p*-value < 0.001; Supplementary Table 2). **b** Difference in the assemblage-based average mushroom color lightness between saprotrophic and ectomycorrhizal fungi (for linear mixed effects model, Supplementary Table 3), and subdivided for each month of the year. Boxplots denote the median (horizontal line) and interquartile range (colored box); whiskers show three times the interquartile range; points indicate values outside this range. Source data underlying panel a are provided in Supplementary Data 1, 2, and 3 and source data underlying panel b are provided in Supplementary Data 4

To test our first objective, we applied a cross-species phylogenetic linear model, based on the Europe-wide data set. We found that the mushrooms of ECM species are significantly darker than those of saprotrophs (Fig. 3a; Supplementary Table 2). Further, based on a linear mixed effect model, we found that the average color lightness of ECM assemblages was significantly darker than saprotrophs (Supplementary Table 3), a pattern consistent across all months of the year (Fig. 3b).

### Color lightness of mushrooms and the thermal environment

To test our second objective, we applied an assemblage-based approach using generalized additive models (GAMs). We used mushroom color lightness as the response and thermal components (mean and variability), seasonality (months), precipitation components (sum and variability), UV-index and relative forest cover as predictor variables, and we accounted for species richness, spatial autocorrelation and monthly resampling of the grid. Species richness of saprotrophs and ECM fungi was equally distributed across Europe (Supplementary Fig. 4).

**Fig. 4:**
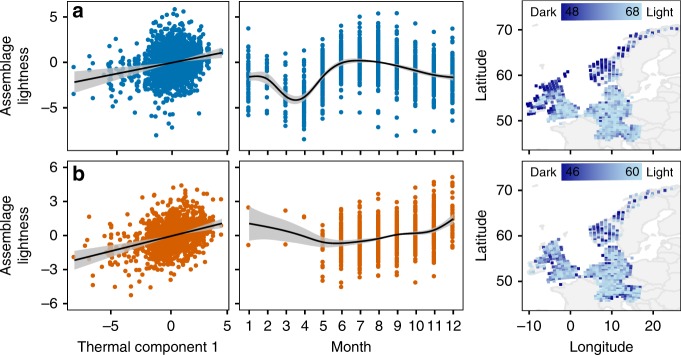
Partial effects of temperature and seasonality on mushroom color lightness. Included here are four orders (Agaricales, Russulales, Boletales, and Cantharellales) of the systematic class Agaricomycetes (Basidiomycota) spanning 3054 species. Partial effects of thermal component 1 (mean temperature) and month on the mushroom color lightness of **a** saprotrophs (blue) and **b** ectomycorrhizal fungi (orange). Maps show mushroom color lightness across Europe. Maps were created using the R package ggplot2 with the function borders. Slopes are estimates from generalized additive models (GAMs) with standard deviations (Table 1 for GAM statistics). Source data are provided in Supplementary Data 4

We found that mushroom color lightness of saprotrophic and ECM assemblages was mainly driven by mean temperature (thermal component 1) and seasonality (Table 1). The relationship between color lightness and mean temperature was significantly positive (SAP: *F*-value = 13.28, ECM: *F*-value = 26.90, Table 1; Fig. 4), meaning that assemblages in cold environments were characterized by a greater proportion of species with dark mushrooms (see also the mapped latitudinal gradient, Fig. 4). Across Europe, assemblages of saprotrophs were significantly darker in colder seasons of the year (spring, fall, and winter), whereas assemblages of ECM fungi showed the opposite pattern (SAP: *F*-value = 75.54; ECM: *F*-value = 26.60, Fig. 4a; Table 1). We also found a significant effect of precipitation sums (precipitation component 1) on mushroom color lightness of saprotrophs (*F*-value = 7.04) but no significant effects on ECM fungi (Supplementary Fig. 5; Table 1). The model of saprotroph mushroom color lightness showed a higher explained variance (*R*² = 0.48) than the ECM model (*R*² = 0.28, Table 1). Further, these results remained consistent under three null models accounting for various potential sampling biases, such as uneven species richness, species frequency or both (Supplementary Fig. 6; Table 1).

**Table 1:** Testing the theory of thermal melanism for mushrooms

	Mushroom color lightness		Independent swap		Richness		Frequency	
	*F*	*p*	*F*	*p*	*F*	*p*	*F*	*p*
*Saprotrophic*								
Thermal comp. 1	**13.28**	**<** **0.001**	**19.74**	**<** **0.001**	**13.31**	**<** **0.001**	**23.10**	**<** **0.001**
Thermal comp. 2	1.88	0.115	1.09	0.426	1.88	0.116	1.37	0.328
Seasonality	**75.54**	**<** **0.001**	**49.67**	**<** **0.001**	**76.11**	**<** **0.001**	**67.53**	**<** **0.001**
Precipitation comp. 1	**7.04**	**<** **0.001**	**7.54**	**<** **0.001**	**7.02**	**<** **0.001**	**7.90**	**<** **0.001**
Precipitation comp. 2	3.27	0.071	**4.21**	**0.040**	3.13	0.077	**4.62**	**0.031**
Relative forest cover	0.03	0.861	1.13	0.286	0.03	0.869	0.06	0.804
UV index	0.03	0.993	0.78	0.541	0.03	0.993	0.87	0.352
Species number	**4.27**	**0.003**	0.77	0.406	**4.31**	**0.003**	**2.67**	**0.025**
Space	**4.14**	**<** **0.001**	**3.46**	**<** **0.001**	**4.03**	**<** **0.001**	**4.22**	**<** **0.001**
Adj. *R*^2^	0.48	0.45	0.48	0.50				
*Ectomycorrhizal*								
Thermal comp. 1	**26.90**	**<** **0.001**	**10.31**	**<** **0.001**	**25.48**	**<** **0.001**	**12.93**	**<** **0.001**
Thermal comp. 2	1.02	0.336	0.85	0.561	1.02	0.344	1.38	0.252
Seasonality	**26.60**	**<** **0.001**	**19.24**	**<** **0.001**	**26.89**	**<** **0.001**	**28.10**	**<** **0.001**
Precipitation comp. 1	0.19	0.666	0.05	0.819	0.15	0.696	0.28	0.600
Precipitation comp. 2	2.15	0.143	0.86	0.354	2.14	0.143	1.82	0.178
Relative forest cover	0.01	0.927	0.03	0.864	0.00	0.946	0.04	0.838
UV index	1.78	0.118	1.28	0.260	1.76	0.121	1.22	0.288
Species number	1.28	0.196	2.19	0.162	1.33	0.199	3.10	0.066
Space	**3.14**	**<** **0.001**	**1.93**	**0.014**	**3.13**	**<** **0.001**	**2.05**	**0.006**
Adj. *R*^2^	0.28	0.31	0.28	0.32				

We present effects (*F*-values) of the mushroom color lightness in response to thermal component 1 (overall temperature means), thermal component 2 (temperature variability), seasonality (month), precipitation component 1 (overall precipitation sums), precipitation component 2 (precipitation variability), relative forest cover and UV index, using log_10_-transformed species number and space (latitude and longitude) as co-variates. The grid number was further used as a random effect. Effect sizes are presented as *F*-values (*F*) and significant effects (*p*-value < 0.05) are emboldened. The assemblage calculation was based on a presence/absence community matrix of 3054 mushroom-forming fungi (1401 ectomycorrhizal; 1653 saprotrophic species). Further, results based on standardized effect sizes using three null models (independent swap, richness, frequency) are shown. For partial effects plots see Fig. 4 and Supplementary Fig. 5. Source data are provided in Supplementary Data 4

In addition to the large-scale European analysis, we used a standardized local-scale and long-term dataset from Switzerland^27^ (32 years of weekly mushroom counts), to test seasonality effects on color lightness, independent of the latitudinal gradient present in the European dataset. Both presence/absence and abundance-weighted measures of the assemblages of saprotrophs consistently showed darker mushroom color in colder months of the year (Supplementary Fig. 7; Supplementary Table 4). We found a stronger effect for the abundance-weighted analysis, indicating a higher proportion of the number of fruit bodies of dark-colored species in cold seasons relative to light-colored species. In contrast to the peak of saprotroph mushroom color lightness in summer months, ECM mushroom color lightness decreased linearly across the fruiting season from spring (May) to late autumn (November) (Supplementary Fig. 7).

Finally, species may retain traits due to shared evolutionary history and are statistically not independent in tests of how the environment affects the color lightness trait^36^. Therefore, we applied a phylogenetic regression in addition to the assemblage-based approach for the European data set^37^. We modeled species lightness as a function of the grid-based mean environmental variables of each species (see “Statistical analyses” in the Methods section for details). We tested various models of trait evolution and 100 alternative trees to account for phylogenetic uncertainty (phylogenetic linear models). We also applied an additional linear mixed model (LMM) with a random effect of genus, to reduce the effective number of degrees of freedom. We found significant positive effects of mean temperature on species color lightness (phylogenetic regression and LMM, Supplementary Tables 5, 6), supporting the results based on the assemblage-based approach (GAM models).

### Effects of climate change on mushroom color lightness

Since temperature has increased by ~1 °C on average in Europe across the last century^38^, we further tested whether the mushroom color lightness has already changed due to increased temperatures. At the temporal scale of our study (1970–2010), the average warming of 0.7 °C has not yet significantly affected mushroom color lightness for assemblages of saprotrophic, nor ECM fungi (Supplementary Fig. 8, linear model: saprotrophs, *R*² = –0.002, *t*-value = 0.576, *p*-value = 0.565; ECM fungi, adj. *R*² = –0.003, *t*-value = 0.076. *p*-value = 0.939).

### Phylogenetic signal of color lightness

**Fig. 5:**
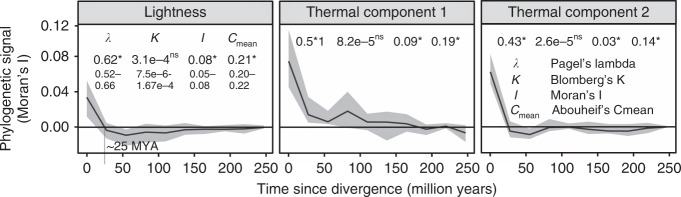
Phylogenetic signal of species mushroom lightness and thermal components. The phylogenetic signal was calculated as four different measures, and graphically displayed based on Moran’s *I*(phylogenetic correlogram). Species occupying at least 10 grid cells were used. The confidence interval (CI), indicated by shading, was based on 99 bootstraps. Significant phylogenetic signal was detectable only over very short phylogenetic distances, i.e., between closely related taxa (CI above/below null line). To estimate overall phylogenetic signal, we used (from left to right): Pagel’s lambda (*λ*), Blomberg’s *K*, Moran’s *I *and Abouheif’s *C*_mean._ The measures of phylogenetic signal show a low signal except for Pagel’s lambda, which displayed medium-high values. Based on simulations, Münkemüller et al.^71^ showed that Moran’s *I* and Abouheif’s *C*_mean_ of 0.1 and 0.2 each, indicate a strength of Brownian motion of approximately 0.3 (on a scale from 0 to 1). Significance of the overall phylogenetic signals was tested based on 99 randomizations. Phylogenetic signal for lightness was further tested for 100 alternative trees and the range of values is given below the lightness values for the maximum likelihood phylogeny. For other environmental variables see Supplementary Fig. 9. Source data are provided in Supplementary Data 1, 2, and 3

To gain an understanding of the evolution of color lightness we explored the phylogenetic signal using various methods. We found a low phylogenetic signal for lightness (Fig. 5 and Supplementary Fig. 9), but a slight significant increase in the phylogenetic signal towards the tips of the phylogeny (~25 million years). We further explored phylogenetic signal in color at the genus level, using the widespread and species-rich genus *Entoloma*. This revealed lightness shifts on very small taxonomic scales, and a positive color lightness response with macroclimatic and seasonal temperature conditions (Supplementary Fig. 10).

### Experimental heating of mushrooms with different color

Finally, we experimentally tested whether mushrooms with dark-colored caps heat up more rapidly than light-colored mushrooms, using two cultured variants of *Agaricus bisporus* (Supplementary Fig. 11). Artificial solar radiation resulted in an increased warming of the dark-colored vs. light-colored mushroom caps. This difference became stronger over time. Using 12 replicates per color, we found that increased warming led to a significant average difference of 1.2 °C after 5 min of artificial solar radiation (Supplementary Fig. 11).

## Discussion

Based on a large-scale European data set, we have demonstrated that dark-colored mushroom assemblages are more prevalent in colder areas and that the thermal environmental variables were the most important predictors in the models. The responses of both saprotrophic and ectomycorrhizal mushroom assemblages to the thermal spatial macroclimate followed the theory of thermal melanism. However, analyzing the seasonal dimension of the thermal melanism theory provided new insights into the response of different fungal lifestyles. While the seasonality analysis provided further support for the thermal melanism theory for saprotrophic fungi, it did not yield support for ectomycorrhizal fungi. Interestingly, mushrooms of ectomycorrhizal species are on average darker than those of saprotrophs, which also translated into mushroom assemblages: ectomycorrhizal fungal mushroom assemblages are likewise darker throughout the season compared to saprotrophs.

We first tested for the difference in color lightness between the nutritional modes. Mushroom fruit bodies of both nutritional modes—saprotrophic and ectomycorrhizal—experience similar constraints and opportunities when developing in similar environments. We speculate that pigments in fruit bodies of both nutritional modes evolved to increase reproductive fitness (e.g., reproductive success^12^) since pigmentation can serve numerous functions (see Discussion section below). The production of many pigments is energetically costly^35^, which might be the key to explaining the observed difference between fruit bodies of fungi with these different lifestyles. We suggest that our observation that ectomycorrhizal fungi evolved darker mushroom phenotypes than saprotrophs (Fig. 3) is a result of increased carbon availability provided by their mutualistic plant partners, which might offset the costs of pigment production^39^. Thus, our findings suggest that transitions from the saprotrophic to the mutualistic lifestyle may have promoted mushroom-darkening. Indeed, our findings are consistent with those of previous studies demonstrating significant differences in morphological traits of the fruit bodies of fungi with these different nutritional modes. For example, mutualistic fungi have larger fruit bodies as well as larger and more ornamented spores compared to saprotrophs^29,30^. Furthermore, mycorrhizal fungi tend to produce larger fruit bodies with a total reproductive biomass greater than that produced by saprotrophs^29,33^, requiring a higher carbon investment. For ectomycorrhizal fungi, carbon is available in excess rather than being obtained by the costly production of enzymes by decomposer fungi^39^. It has therefore been suggested that the evolution of the mycorrhizal lifestyle in mushroom-forming fungi increased their reproductive fitness by receiving carbon from the symbiotic host plant, which is consistent with mutualism theory^29,30,40^. If the production of pigments is costly and pigments are correlated with reproductive fitness, we would expect that the mycorrhizal lifestyle could afford a higher level of mushroom pigmentation compared to the saprotrophic lifestyle. Nevertheless, fungal carbon allocation may underlie a trade-off between investment into mycelium and reproductive structures in both lifestyles. This trade-off however, might be less strict for ectomycorrhizal fungi given their higher carbon availability due to the mutualistic relationship.

However, mutualistic relationships not only provide opportunities but also constraints. For example, environmental selection on ECM fungi can be affected by the hosts’ biology and ecology via at least two possible mechanisms: (1) ECM species occur in darker habitats owing to their symbiosis with tree species that form forests; and because forests are darker and thus colder than open fields or grasslands, at least during the daytime, ECM species might have evolved darker mushroom phenotypes to enhance warming by solar radiation. This potential mechanism is, however, currently difficult to assess because habitats are still poorly characterized for most species. (2) ECMs tend to produce fruit bodies towards late autumn (September, October), when the host tree photosynthesis and carbon allocation to ECM species is changing^28,41^. Late fruiting coincides with lower temperatures, which could also lead to stronger selection for darker mushrooms among ECM species. By contrast, saprotrophs are probably less influenced by temporal constraints on fruiting^28,41^, and thus avoid very low temperatures. The second mechanism is supported by our local seasonal analysis showing darker ECM mushroom assemblages in autumn (Supplementary Fig. 7), however, not by the seasonal analysis across Europe (Fig. 4). Nonetheless, ECM mushroom color lightness is darker in every month of the year compared with saprotrophs, which might allow thermoregulation (Fig. 3b). Nonetheless, the observed contrasting results between the datasets need further attention in future studies.

The thermal environment varies not only spatially but also seasonally. Previous tests of the theory of thermal melanism focused on macroclimatic gradients^5–7,10^. We not only considered macroclimate gradients but further included a temporal-seasonal analysis as temperature varies considerably within the year. We expected that mushroom color is darker in spring and fall and lighter in summer. The seasonal analysis of the patterns was consistent with the theory of thermal melanism based on the European and local data set for saprotrophs but not for ectomycorrhizal assemblages. Moreover, for ectomycorrhizal assemblages the pattern differed also between the local and the European data set (Fig. 4 and Supplementary Fig. 7). We can only speculate about the mechanism: if a darker phenotype generally allows better thermoregulation in cold seasons, ECM fungi with their generally darker phenotype (as shown here: Fig. 3a, and above discussion) might be additionally selected by other factors. For example, pigments might help mitigate the mechanical and chemical stress, such as caused by radiation or drought, by providing the ability to sequester metal ions and scavenge free radicals^42^. Moreover, selection for specific pigmentation phenotypes in a given environment might also help avoid attacks of predators (aposematism, camouflaging, and mimicry^11^) or in contrast, to attract potential vectors of spores^31^, as suggested for bioluminescence^43^. Mushroom color has been suggested as a warning to herbivores^13^, but there is no supporting evidence for this. More studies are clearly needed and we provide a conceptual overview of hypotheses in Supplementary Fig. 12.

The main focus of our study was the response of mushroom color lightness to the thermal environment. However, studies addressing the color lightness of insects have often found significantly darker assemblages in environments with high humidity or UV radiation^5–7^, indicating that pigmentation of ectothermic organisms might have functions other than thermal melanism. By including additional variables in our models that could affect mushroom coloration, we were able to test the relative importance of thermal environment and other plausible mechanisms. The thermal variables had the strongest effects on mushroom color lightness of saprotrophic and ECM fungi in our study (Table 1), but there was also a significant negative relationship between precipitation (precipitation component 1) and color lightness of mushrooms of saprotrophic fungi (Supplementary Fig. 5; Table 1). A recent study found a negative correlation between humidity and Lepidoptera color lightness^7^. Humid habitats coincide with a higher pathogen (bacterial, fungal) pressure^44^, and melanin-based dark pigments protect insects against pathogens^45^. There is no evidence for this type of protection by mushroom pigments^42^, but fungal pigments are associated with antimicrobial activities^42^. Further, mechanical damage, including heavy precipitation, of light-colored, fragile, soft-fleshed mushrooms might be ameliorated as pigments can stabilize cell walls (Supplementary Fig. 12). These additional factors could help explain why ectomycorrhizal fungi, with generally darker phenotypes compared to saprotrophic fungi, did not show a similar response to seasonality (Fig. 3).

Many studies addressing the theory of thermal melanism in insects have shown that high UV radiation can select for darker assemblages^1,5,7^. Melanin also reduces UV-radiation-induced stress in fungal mycelium and spores^42^ and might therefore be expected to affect mushroom color lightness. However, we did not find a significant relationship between mushroom color lightness and UV index (Supplementary Fig. 5; Table 1). Indeed, we found that assemblages of saprotrophic fungi were darker in spring, fall and winter and lighter in the warmest months (Fig. 4), when UV would be highest. These results together strongly suggest that UV radiation is not a significant driver of mushroom color lightness at the scale of our study. One explanation for the contrasting responses of (saprotrophic) fungi and insects to UV radiation^5,7^ might be that mushrooms are more ephemeral (hours to days)^17^ than butterflies or dragonflies (weeks to months^46^), thus, the UV damage might be considerably less.

Here we followed a macroecological approach to reveal the relationship between mushroom color lightness and the thermal environment across a large spatial scale. However, effects of large-scale environmental gradients might be mediated by local scales factors. From local studies we know that canopy cover can strongly affect microclimate conditions^20,21^. Both mean and variability of temperature and precipitation within an area can be influenced by canopy cover (proportion of forest cover). However, we did not detect significant effects of relative forest cover (Table 1). Explanations might include that relative forest cover does not fully represent temperature variability within a grid cell, or perhaps tree density is more important than forest cover. Future studies using a smaller grain size could help to tease apart the mechanisms of how climate and vegetation may interact to influence fungal traits.

We were further interested in whether mushroom color lightness has responded to recent climate warming. The main finding of our study is that mushroom color lightness is darker in cold climates (Fig. 4; Table 1). Climate change could have negative effects on mushroom biogeographical distributions e.g., due to overheating^47^. However, even though the trend in mushroom color lightness over time was slightly positive for both saprotrophic and ECM assemblages, the change was not significant (Supplementary Fig. 8). Possible explanations for the lack of relationship between climate change and mushroom lightness may be that: (1) species occurrences have remained stable but their abundances have changed, which we are not able to track given that our dataset consists of presence/absence data; (2) species occurrences are stable at the 50 × 50 km grid scale, as they can disperse and find suitable habitats within this area. For example, it is well known that many species move towards higher altitudes due to climate warming^48^; (3) dark-colored species might still persist by occupying a suitable “cold” niche within a region^49^. Cold pockets sometimes allow plant species to remain in the same elevation in our warming climate^50^. Further studies are hence needed to quantify changes in abundance on a larger scale, using fine-grained resolution. A local study in Spain demonstrated a drought-induced reduction of sporocarps and species richness since 1995^51^. This suggests that global warming affects mushroom fruiting. A warmer environment might allow light-colored species to colonize and dark-colored species might go locally extinct if physiological constraints are limiting their metabolism (e.g., overheating) and may be prone to colonize higher altitudes/latitudes. However, our results suggest that a shift in the community composition (change of occupancy pattern, not abundance) due to global warming, as a result of pigmentation, is likely a longer-term process. Within insects such shifts have already been reported^6,52^. Insects might respond more rapidly to climate change than fungi, due to a higher active mobility (non-sessile organisms), compared with sessile fungi, where range shifts may be slower.

We found a low phylogenetic signal for lightness (Fig. 5), suggesting that lightness of mushroom-forming fungi is not strongly retained. The phylogenetic signal only increased slightly but significantly towards the tips of the phylogeny (~25 million years), indicating conserved evolution below the genus level (Fig. 5). Studies addressing phylogenetic signal in color traits are rare, but low phylogenetic signal has been found in plant fruit colors^53^. Supporting the general finding of low phylogenetic signal, the widespread and species-rich genus *Entoloma* demonstrated that lightness shifts also occurred on very small taxonomic scales, resulting in a positive color lightness response with temperature and seasonality, even within this genus (Supplementary Fig. 10). Taxonomically well-sampled fine-scale phylogenomic studies can further elucidate the evolutionary basis of the color trait.

In conclusion, our results provide the first evidence that a morphological trait—mushroom color—contributes to structuring fungal communities at large spatial scales (Europe). We demonstrated that dark-colored mushrooms heat up more rapidly than light-colored mushrooms and that dark-colored assemblages are more prevalent in colder areas. These patterns thus yield further support extending the theory of thermal melanism from the animal to the fungal kingdom and within this kingdom from unicellular^14^ to multicellular fungi. We hypothesize that fungi with dark-colored mushrooms are at an advantage in cold climates via increased reproductive success. The low phylogenetic signal of color lightness supports the interpretation as adaptive selection by the thermal environment. However, the temporal-seasonal dimension of the theory of thermal melanism was supported for saprotrophic fungi but not ectomycorrhizal fungi, suggesting: (i) lifestyle-dependent support for the theory of thermal melanism; (ii) additional functions of pigmentation beyond thermal regulation; (iii) that tests of the theory of thermal melanism should include both spatial and seasonal thermal analyses, along with other functions of pigmentation. The study of color has a long tradition in animals and plants, but our study strongly suggests the need for more research efforts to understand the biology of colors in the fungal kingdom.

## Methods

### European and local fungal species distribution dataset

This study utilized data from a component of the ClimFun meta-database, a source of unified, multi-source data that originated from many independent data repositories of fungal fruiting records across Europe^22^. A detailed description and figures of the raw data can be found in Andrew et al.^22^. We used data from eight of nine countries with substantial numbers of records across the time span of 1970–2010. The composition of fungal assemblages was summarized within 50 km × 50 km grid cells, utilizing the UTM coordinate system (zone 32). This seems an adequate grain size for multi-source data, and the spatial extent of our study, and was often used in similar macroecological analysis^6,7,10,54^. The resulting community matrix consisted of 5831 species and 743 grid cells. We further used a local scale dataset: the data set is based on weekly mushroom counts (total 115.417 fruit bodies) between 1975 and 2006. Five plots of 300 m^2^ were set up in the fungus reserve La Chanéaz in western Switzerland in a temperate forest on an area of 75 ha. The dataset is standardized as it does not include an altitude gradient. We summed weekly records of all plots to obtain monthly values for each year and removed species which were found in less than three months across all years. Further details can be found in Büntgen et al.^27^. We coded the main genus-level nutritional mode (saprotrophic or ectomycorrhizal) for each species based on literature^55,56^. The nutritional modes were equally distributed across the latitudinal gradient of the dataset (Supplementary Fig. 4).

### Environmental data

Our study focused on the relationship between mushroom color lightness and temperature on a macroecological scale. Since the relationship between mushroom color lightness and temperature might additionally depend also on other potential functions of pigmentation, we also considered other environmental variables in our models. Among these variables, we expected that precipitation, UV radiation, and relative forest cover (as a proxy for microclimate conditions reflecting e.g., local variation in temperature and moisture) may be particularly important^25^.

### Grid data preparation

For each grid cell we used 21 environmental variables which were extracted using the R package raster^57^. (i) Temperature-related variables (mean annual temperature, mean diurnal range, isothermality, temperature seasonality, maximum and minimum temperature of warmest and coldest month, temperature annual range, mean temperature of wettest, driest quarter and warmest and coldest quarter), were taken from WorldClim^58^; (ii) Precipitation-related variables (annual precipitation, precipitation of wettest, driest, warmest and coldest month, precipitation seasonality, precipitation of wettest and driest month), were also from WorldClim^58^; (iii) UV index was from the NASA website^59^; (iv) Relative Forest cover was from the Forest MAP 2000 dataset^60^. All available environmental variables were gridded at the 50 km × 50 km level. We calculated the 50 km × 50 km grid cell means only within cells containing fungal records based on a 10 × 10 km resolution (subgrids). This was possible for the climate and forest cover data, however, UV index data were only available on 50 × 50 km resolution. Therefore, we explored, whether differences in the raw resolution among predictors might have an influence on the statistical inference. For this purpose, we standardized all environmental variables to the same resolution (50 km × 50 km ignoring a weighting based on fungal records). We then subjected both predictor sets (weighted based on fungal records and unweighted) to a Principal component analysis (PCA) and a comparison revealed: (i) a similar relationship among different predictors; and (ii) the same meaning (loading) within predictors (Supplementary Fig. 13). We, therefore, decided to calculate the mean of each 50 km × 50 km grid using the highest resolution available for the environmental data, to increase accuracy of our models.

For the UV index, we downloaded all available grid data for each month of 2010 and calculated the mean UV index across the 12 months for each grid. As far as we know, no reliable long-term UV radiation data are available at the scale of our study. However, variability of UV radiation for a given locality across years is less pronounced than the variability across localities, indicated by straight and parallel isolines^59^. Solar radiation models show that UV radiation underwent a net change in UV-B radiation of ca. 3% in the time period 1970–2010^61^. Furthermore, these changes through time were spatially consistent across the latitudinal range of our study^59^. Thus, we consider the values of 2010 to be a robust proxy for UV radiation for the temporal scale of our study.

For the relative forest cover per grid cell, each 50 km × 50 km plot covers 4 × 10^6^ pixels of satellite imagery. We obtained the relative forest cover per grid cell as the ratio of forest pixels to non-forest pixels (e.g., grasslands). Note that some grid cells were partly covered by clouds, hampering an exact estimation of forest cover. However, considering clouds to be forest or ignoring clouds resulted in comparable estimates of forest cover. We first gridded relative forest cover on a 10 km × 10 km grid and then calculated the 50 km × 50 km grid means by only considering 10 km × 10 km grids occupied by fungi (see above).

To address and reduce potential biases associated with observational data, we (i) reduced the dataset to mushroom-forming fungi, which are mainly found in the four orders of the systematic class Agaricomycetes (Basidiomycota): Agaricales, Boletales, Russulales, and Cantharellales^17^. This standardized the dataset to a unique fruit body type, characterized by soft-fleshed, above-ground stems and caps. This conspicuous mushroom type attracts most field mycologists. (ii) Because the number of records of a species in a grid cell might reflect collection effort rather than true abundance, we only used the occurrence of each species in each grid cell. The long sampling period, comprising four decades, ensures that these data can be reliably interpreted as estimates of the presence/absence of the species. (iii) To account for possible spatial biases in collection activity, we only included grid cells with a minimum of 25 species occurrences for each nutritional mode in the analyses (resulting in 549 and 522 out of the 743 grid cells for saprotroph and ECM fungi respectively, reducing the dataset mostly in the very north of Norway). Despite this thorough data preparation, spatial biases in collection activity may still bias data interpretation. Thus, we applied null models to account for (1) uneven richness among grids (null model “richness”), (2) uneven sampling probability of species (null model “frequency”), and (3) both factors simultaneously (null model “independent swap”). For a detailed description of the null model approaches, see “Statistical analysis” below. Note that our response of interest is a mean assemblage trait, calculated based on the lightness values of the species in an assemblage. If temperature acts as an environmental filter, the mean color value should reflect temperature conditions on a grid even though sampling effort differs among grids.

To address a possible shift in color lightness with climate change, we divided our 40-year dataset into two time intervals: 1970–1990 and 1991–2010. The latter interval is the time of accelerated climate warming^62^. As temperature data, we used the E-OBS dataset from the EU-FP6 project ENSEMBLES (http://ensembles-eu.metoffice.com) and the data provided in the ECA&D project (http://www.ecad.eu)^63^. We gridded the temperature on our 50 km × 50 km grids by selecting the nearest-neighbor temperature value (from the 1/4 degree temperature data) within a maximum range of 15 km. As for the other environmental variables the 50 km × 50 km grid values were calculated as means based on the finest resolution possible, here thus 25 km × 25 km subgrids with fungal data. For the analyses, we considered only grids with at least 25 species for each nutritional mode in each of the time periods. We reduced the datasets of the two time-intervals to a subset of common grids, resulting in 356 grid cells. The average temperature difference between the second and first time-interval was 0.7 °C and was normally distributed with a range of 0.26 to 1.34 °C. We then calculated the difference in temperature and the difference in mushroom color lightness between the second and first time-interval^6^.

### Color sampling

We recorded three independent variables for the cap color of each species, based on the cylindrical-coordinate representations of points in an HSL color model: hue (e.g., red, blue), saturation (amount/intensity of hue), and lightness (e.g., ranging from light red to dark red). For more details on the color model, see ref. ^64^. Such human vision models have been shown to reliably detect variation in color in the visible range^65^. We used the independent component lightness (L) as the basis to calculate the average assemblage lightness (“mushroom color lightness”) based on the community matrix. We then used the mushroom color lightness as the response variable in our generalized additive models.

We searched relevant websites (general mushroom websites, e.g., mycokey.com, mykoweb.com, 123pilze.de, mushroomobserver.org, mushroomexpert.com, grzyby.pl, pilze-basel.ch, mycodb.fr, mycoleron.fr, mykologie.net, discoverlife.org, tintling.com, hlasek.com, fungipedia.org, Wikipedia, mykologie.net, mycoportal.org; and specialized taxon websites, e.g., mycena.no, cortinarius.org, amanitaceae.org, entoloma.de, inocybe.org, boletales.com) for representative images employing the following quality criteria: (i) experts (among authors) chose images with the best color representation of the mushrooms; (ii) at least half of the cap was visible; (iii) overexposed (flashy) or strongly shaded areas in images were not sampled; and (iv) cap areas with reflections by water drops, earthy dirt, or sticky leaves on the cap were not sampled. For species represented by adequate images, we sampled HEX values using the program “pipette” (Stefan Trost Media, http://www.sttmedia.com/). For all mushroom-forming species from the dataset, we sampled HEX values of nine areas on each mushroom cap, situated on a cross (center, edge, and between center and edge). For species with variable colors, we sampled at least two images. Each of the nine HEX values was then converted into three values (H, S, and L) using the website: http://rgb.to. Thus, each mushroom cap yielded at least nine values for hue, nine for saturation, and nine for lightness. We then calculated the means of H, S, and L separately for each species and used these mean values to characterize species color traits in further analyses. The mean hue for each species was calculated using a circular model (R package circular). Visually, the species lightness sampling corresponded well with the opinions of experts (among co-authors) on mushroom colors (Fig. 2). To further assess the reliability of our color lightness web survey, we (i) tested the color lightness against color lightness retrieved from standardized illustrations (Supplementary Fig. 2B) and (ii) explored the data for a potential latitudinal bias in color lightness (Supplementary Fig. 2C). Previous studies have been entirely based on illustrations^6,10^; however, only a subset of the species used in this study were already illustrated (for further information see caption of Supplementary Fig. 2). We found a high fit between the color lightness of the web survey and the color lightness of the illustrations (*R*^2^ = 0.86, slope not significantly different from 1) and no bias in color lightness with latitude (Supplementary Fig. 2).

### Mega-phylogeny approach

To test for phylogenetic influences on mushroom lightness, and to carry out phylogenetic linear regressions (see “Statistical analyses” below), we applied a mega-phylogeny approach. To optimize the phylogenetic tree, we integrated prior knowledge into the tree inference^66^. For our mega-phylogeny approach we followed the protocol described in Krah et al.^67^. In short, we used five gene regions (28S and 5.8S rRNA, *rpb1*, *rpb2, tef1*), with gene partitioning, that resulted from a partition scheme software^68^, a comprehensive back bone guide tree based on phylogenomic analysis and a column reliability score. We used a guide tree to increase topological accuracy and thus a higher number of species could be added, which would otherwise have no support because of low sequence information. We further conducted 1000 approximate Shimodaira–Hasegawa likelihood ratio tests to assess branching support (SH-aLRT branch support) using RAxML (flag -f J). The final phylogeny consisted of 1,011 ectomycorrhizal and 1046 saprotrophic fungal species (Supplementary Fig. 3). We estimated divergence times of the resulting phylogeny using penalized likelihood as implemented in the R function *chronos*. Although we did not interpret timing of events based on our phylogeny, we time-dated our phylogeny using two calibration points. The branching time estimates fall within the estimates of previous studies^23,26^. To estimate the effect of phylogenetic uncertainty on our interpretations, we repeated phylogenetic analyses with a set of 100 alternative trees. These trees were derived by creating polytomies on nodes with an SH-aLRT branch support value below 80 based on the non-ultrametric ML tree. These multifurcations were then resolved randomly using the function *multi2di* from the R package ape^69^. We then estimated divergence times for each tree following the same calibration protocol as above using *chronos*.

### Phylogenetic signal

We calculated the phylogenetic signal in color lightness and the environmental variables using four indices: Pagel’s lambda (*λ*), Blomberg’s *K*, Moran’s *I*, and Abouheif’s *C*_mean_ using the function *phyloSignal* (R package phylosignal^70^, with 99 randomizations). For a detailed description and simulation-based tests of all four indices, see Münkemüller et al.^71^, who recommended choosing specific methods of phylogenetic signal based on the expected underlying pattern of evolution, however, we did not have an assumption of the evolution of color in mushrooms due to the lack of prior studies. We thus decided to use four measures of phylogenetic signal reviewed by Münkemüller et al.^71^ to enable a higher comparability with future studies.

### Statistical analyses

Based on the monthly grid-by-species community matrix and the species color lightness values, we calculated an average color lightness of assemblages (“mushroom color lightness”) as response variable, where assemblages are defined as the total species composition found within a grid cell and month, aggregated across the years 1970–2010.

To reduce dimensionality in the bioclimatic variables, we applied PCA separately for temperature and precipitation and for lifestyles (Supplementary Fig. 13). One component reflected overall means (e.g., mean annual temperature, mean annual sum of precipitation) and one reflected variability of temperature and precipitation (e.g., seasonality). We call the component reflecting overall mean temperatures “thermal component 1”, the component reflecting mean precipitation sums “precipitation component 1” and the components reflecting temperature and precipitation variability “thermal component 2” and “precipitation component 2”, respectively. We further considered UV index and relative forest cover as described above (see “Grid data preparation” above). Besides the thermal components we also considered “month” as temperature seasonality, however, we retained the term “seasonality” because the variable was not derived from a PCA. We finally checked the collinearity among all environmental variables before fitting the models. Strong collinearity among co-variables in a model can cause spurious effects and we therefore considered only environmental variables in our models with pairwise correlation coefficients |*r*| < 0.6 as a conservative threshold^72^. However, according to this criterion, we observed strong collinearity only for the pairwise comparison between precipitation component 2 and the thermal component 2 (Supplementary Table 7). A comparison of the models excluding and including precipitation component 2 yielded similar results. We therefore decided to include all co-variables for the main models of our study.

To test our hypotheses, we fitted four models: three models based on the European dataset (3054 species) and one on the local-scale dataset (312 species). (1) A linear mixed effects model with the mushroom color lightness as response and the nutritional mode as predictor and grid cell as random effect. (2) A generalized additive model with mushroom color lightness as the response and environmental variables as predictors, along with species number (log_10_-transformed). To account for spatial autocorrelation, we added geographical latitude and longitude as covariates (“s(longitude, latitude)”). We further added grid cell as a random effect (“s(grid, bs = “re”)”). (3) A model as in (2) but with standardized effect sizes as responses, calculated from three different null models. These null models were used to compute standardized effect sizes (SES): “richness” (randomizes community data matrix within grids; maintains sample species richness), “frequency” (randomizes the community data matrix within species; maintains species occurrence frequency), and “independent swap” (randomizes community data matrix with the independent swap algorithm^73^, maintaining species occurrence frequency and grid species richness) available in the function *randomizeMatrix*. We randomized the community matrix 100 times (and 1000 swaps each) and computed the expected color lightness mean for each grid cell and the expected standard deviation. The standardized effect size (SES) was computed by subtracting the expected mean from the observed mean, and dividing by the standard deviation (SD, calculated from 100 values of the randomization). (4) Finally, for the local-scale data set, we applied a generalized additive model with mushroom color lightness as response and the month as predictor variable. We calculated the linear mixed effects model using the function *lmer* from the R package lmerTest^74^ and the generalized additive models using the function *gam* from the R package mgcv^75^.

Species are statistically non-independent as they share common evolutionary history^36^. Therefore, we fitted three additional models on the species level, considering those species with phylogenic data (2057 species): (1) A phylogenetic regression model with species lightness as the response and nutritional mode (ectomycorrhizal vs. saprotrophic) as a binary predictor variable. (2) We furthermore calculated a phylogenetic regression model with species lightness as the response and species environmental means (niche positions) as predictors. Besides testing the overall effect of the environmental variables on species lightness, we furthermore tested the interaction of the species’ nutritional mode. We first calculated the mean environmental niche position for each species. We restricted this analysis to those species that occurred on ≥10 grid cells, in order to obtain a robust mean value that resulted in 1662 species. Considering those species that occurred on ≥5 and ≥15 grid cells produced similar results. Before we fitted the model, we checked the collinearity among the environmental variables as outlined above. Here however, we observed several pairwise correlations with exceeding the threshold |*r*| ≥ 0.6 (Supplementary Table 7). Since linear models are particularly sensitive to collinearity^76^, we decided to remove both precipitation components, UV and relative forest cover and considered only the thermal components which are the main focus of our study. We repeated the phylogenetic regression models using 100 alternative phylogenetic trees to assess the effect of phylogenetic uncertainty. We used phylogenetic regressions using the function *phylolm* from the R package phylolm^77^ and used three evolutionary models of trait evolution: Brownian motion (BM), Ornstein-Uhlenbeck (OU) with random root and Pagel’s lambda (*λ*). We then compared the models using Akaike’s information criterion (AIC). Finally, we fitted a linear mixed effects model with the same structure as described in (2) but instead of using the complete phylogenetic tree, we added the genus as random effect in order to avoid incorrect degrees of freedom if clades share the same trait owing to common descent. According to Grafen^37^, each radiation (e.g., radiation of ectomycorrhizal fungal clade) should be treated as an independent data point with one degree of freedom (“radiation principle”). We used the genus level because we found increased phylogenetic signal mainly within genera (Fig. 5).

### Experimental effect of radiation on mushroom temperature

To test how mushroom cap lightness affects their temperature dynamics, we used two cultured breeding variants (brown = dark and white = light) of *Agaricus bisporus*^78^. All mushroom individuals were of similar size and fresh weight. We first cooled mushroom caps to ca. 12 °C in a refrigerator and measured the initial cap temperature. We then placed caps beneath a solar lamp (Lucky Reptile Bright Sun UV Desert, 50 W), which we positioned at a distance of ca. 40 cm to create ca. 30 °C on the cap surface. We measured the cap surface temperature at regular intervals over time using an infrared thermometer (removing the caps from the solar lamp for measurement). We alternated the order of measurement of dark and light caps. For a more robust statistical test of the difference in warming between light-colored and dark-colored caps, we then measured 12 dark-colored and 12 light-colored caps before and after solar lamp exposure and measured temperatures after 5 min.

### Reporting summary

Further information on research design is available in the Nature Research Reporting Summary linked to this article.

## Supplementary information

Supplementary Information

Reporting Summary

Description of Additional Supplementary Files

Supplementary Data 1

Supplementary Data 2

Supplementary Data 3

Supplementary Data 4

Supplementary Data 5

Supplementary Data 6

Supplementary Data 7

Supplementary Data 8

## Data Availability

All data analyzed during this study are included in this published article and its supplementary information files.
