# Supplementary material for: European mushroom assemblages are darker in cold climates
Source: Nat Commun. 2019 Jun 28;10:2890. doi: 10.1038/s41467-019-10767-z (PMC6599080; doi:10.1038/s41467-019-10767-z)
Supplement: Supplementary file 1 — Supplementary Information [file 41467_2019_10767_MOESM1_ESM.pdf]

Supplementary Information

European mushroom assemblages are darker in cold climates

Krah *et al*.

This PDF file includes:

Supplementary Figures 1-13

Supplementary Tables 1-7

Supplementary References

Supplementary Figures and Tables

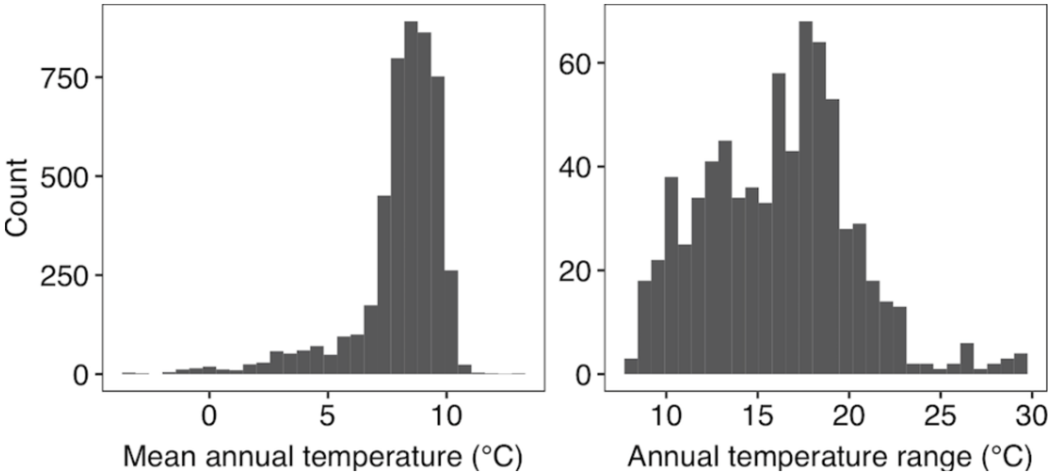

Supplementary Figure 1. Histograms of temperature of the grids in the European dataset. Displayed are mean annual temperature values and the annual temperature ranges (maximum – minimum monthly temperature) of each grid cell used in this study.

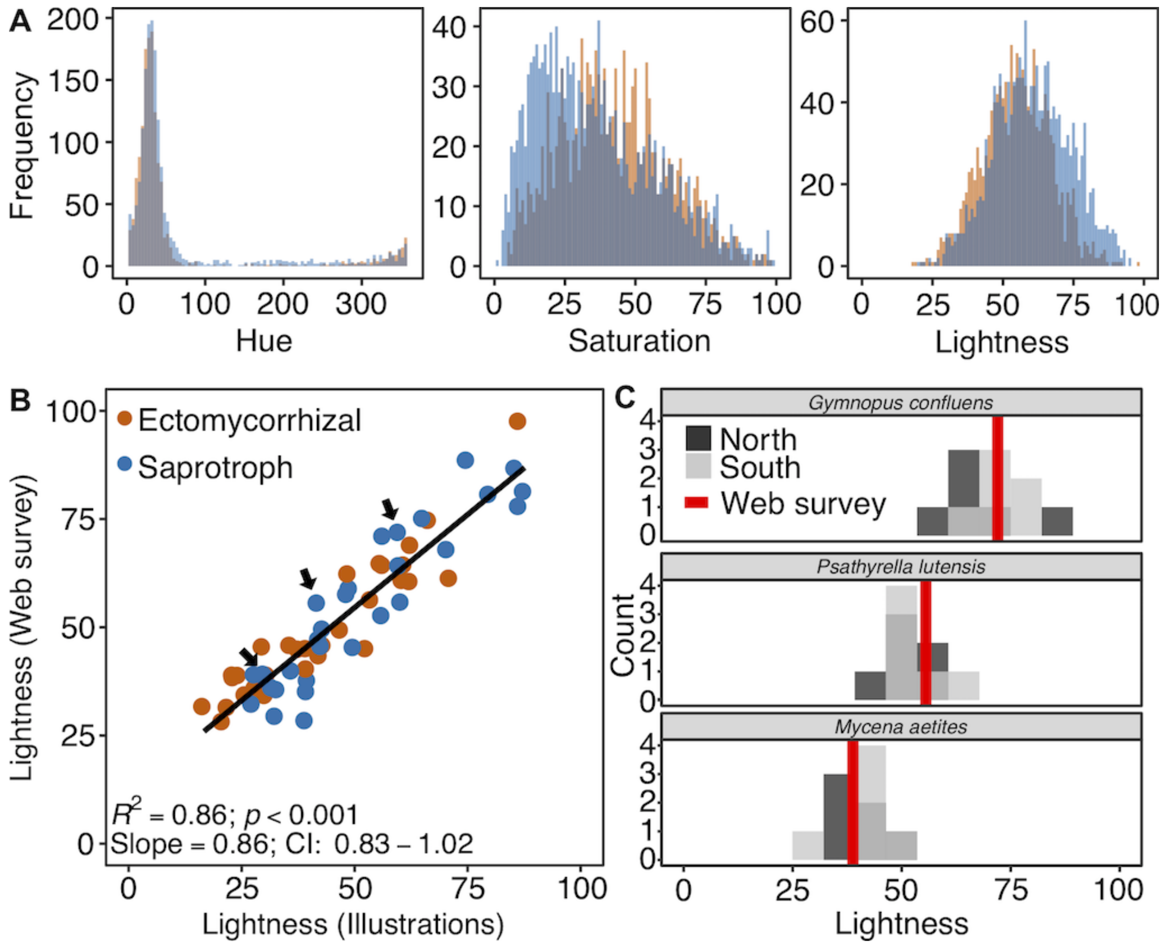

Supplementary Figure 2. Histograms of mushroom colors and data reliability. (**A**) Histograms based on ectomycorrhizal and saprotrophic species (3,054 species). Both, saprotrophs (blue) and ectomycorrhizal fungi (orange), show a hue peak in the interval 1–80 degree, which comprises the colors red, orange, and yellow (note that this includes brown colors). Ectomycorrhizal fungi have on average darker fruit bodies than saprotrophic fungi (Fig. 3A, Supplementary Table 2). Note that overlapping areas are displayed in darker blue. (**B**) Lightness based on web survey (our color sampling approach, see 'Color sampling' in Methods) vs. lightness based on standardized illustrations [1–4]. Illustrations are standardized in terms of geographic and environmental variability of lightness intensity. We randomly selected 10 species of each of three lightness categories (dark, <40; medium, 40-60; and light, >60, see Fig. 2) and nutritional modes (ectomycorrhizal and saprotroph) from our dataset, resulting in a total of 60

species. We then generated lightness values for the same species based on illustrations [1–4]. Note that standardized illustrations are only available for a limited subset of our species list. Goodness of fit (R^2) and *p*-value (*p*) were estimated using linear models. We further tested for proportionality by testing whether the estimated slope differed from a slope of one, using reduced major axis**.** Arrows indicate species used in the analysis in (C). (**C**) Lightness based on web survey in northern latitude (>~58 latitude) vs. southern latitudes (<~50 latitude) based on three saprotrophic species (dark, <40; medium, 40-60; and light, >60) with each 6 individuals from north and south. The red line indicates the lightness value of the web survey (our color sampling approach, see 'Color sampling' in Methods) used in our analysis. Overlapping areas of the northern and southern distribution are shown in an intermediate gray. Source data underlying panel **A** are provided in Supplementary Data 1, 2 and 3. Source data underlying panels **B** and **C** are provided in Supplementary Data 5.

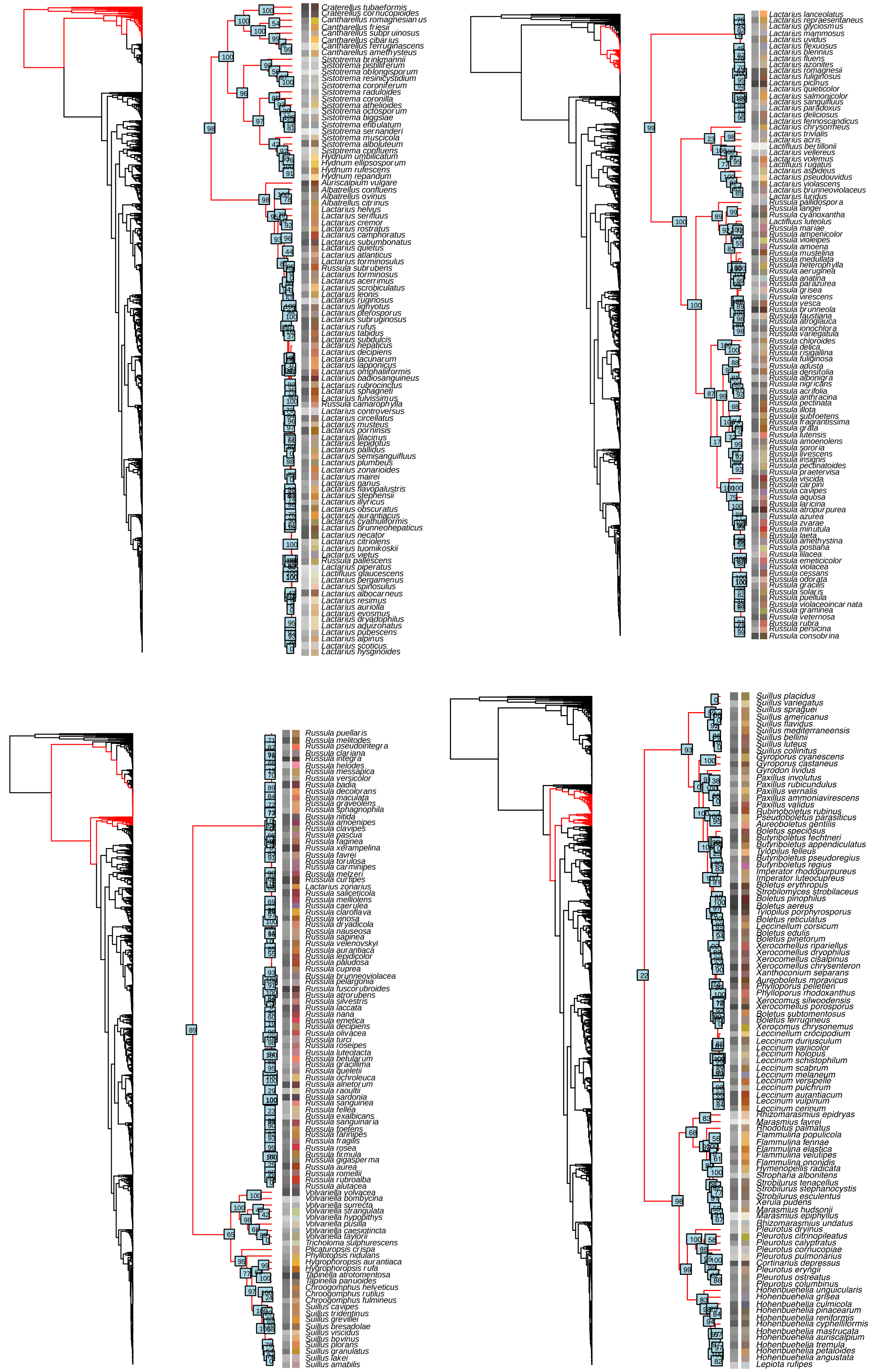
Craterellus tubaeformis
Craterellus cornucopioides
Cantharellus romagnesianus
Cantharellus friesii
Cantharellus subpruinosus
Cantharellus cibarius
Cantharellus ferruginascens
Cantharellus amethysteus
Sistotrema brinkmannii
Sistotrema pistilliferum
Sistotrema oblongisporum
Sistotrema resinicystidium
Sistotrema coroniferum
Sistotrema raduloides
Sistotrema coronilla
Sistotrema athelioides
Sistotrema octosporum
Sistotrema biggsiae
Sistotrema efibulatum
Sistotrema sernanderi
Sistotrema muscicola
Sistotrema alboluteum
Sistotrema confluens
Hydnum umbilicatum
Hydnum ellipsosporum
Hydnum rufescens
Hydnum repandum
Auriscalpium vulgare
Albatrellus confluens
Albatrellus ovinus
Albatrellus citrinus
Lactarius helvus
Lactarius serifluus
Lactarius cremor
Lactarius rostratus
Lactarius camphoratus
Lactarius subumbonatus
Lactarius quietus
Lactarius atlanticus
Lactarius torminosulus
Russula subrubens
Lactarius torminosus
Lactarius acerrimus
Lactarius scrobiculatus
Lactarius leonis
Lactarius ruginosus
Lactarius lignyotus
Lactarius pterosporus
Lactarius subruginosus
Lactarius rufus
Lactarius tabidus
Lactarius subdulcis
Lactarius hepaticus
Lactarius decipiens
Lactarius lacunarum
Lactarius lapponicus
Lactarius omphaliiformis
Lactarius badiosanguineus
Lactarius rubrocinctus
Lactarius sphagneti
Lactarius fulvissimus
Russula camarophylla
Lactarius controversus
Lactarius circellatus
Lactarius musteus
Lactarius porninsis
Lactarius lilacinus
Lactarius lepidotus
Lactarius pallidus
Lactarius semisanguifluus
Lactarius plumbeus
Lactarius zonarioides
Lactarius mairei
Lactarius nanus
Lactarius flavopalustris
Lactarius stephensii
Lactarius illyricus
Lactarius obscuratus
Lactarius aurantiacus
Lactarius cyathuliformis
Lactarius brunneohepaticus
Lactarius necator
Lactarius citriolens
Lactarius tuomikoskii
Lactarius vietus
Russula pallescens
Lactarius piperatus
Lactifluus glaucescens
Lactarius pergamenus
Lactarius spinosulus
Lactarius albocarneus
Lactarius resimus
Lactarius auriolla
Lactarius evosmus
Lactarius dryadophilus
Lactarius aquizonatus
Lactarius pubescens
Lactarius alpinus
Lactarius scoticus
Lactarius hysginoides
Lactarius lanceolatus
Lactarius repraesentaneus
Lactarius glyciosmus
Lactarius mammosus
Lactarius uvidus
Lactarius flexuosus
Lactarius blennius
Lactarius fluens
Lactarius azonites
Lactarius romagnesii
Lactarius fuliginosus
Lactarius picinus
Lactarius quieticolor
Lactarius salmonicolor
Lactarius sanguifluus
Lactarius paradoxus
Lactarius deliciosus
Lactarius fennoscandicus
Lactarius chrysorrheus
Lactarius trivialis
Lactarius acris
Lactifluus bertillonii
Lactarius vellereus
Lactarius volemus
Lactifluus rugatus
Lactarius aspideus
Lactarius pseudouvidus
Lactarius violascens
Lactarius brunneoviolaceus
Lactarius luridus
Russula pallidospora
Russula langei
Russula cyanoxantha
Lactifluus luteolus
Russula mariae
Russula amoenicolor
Russula violeipes
Russula amoena
Russula mustelina
Russula medullata
Russula heterophylla
Russula aeruginea
Russula anatina
Russula parazurea
Russula grisea
Russula virescens
Russula vesca
Russula brunneola
Russula faustiana
Russula atroglauca
Russula ionochlora
Russula variegatula
Russula chloroides
Russula delica
Russula risigallina
Russula fuliginosa
Russula adusta
Russula densifolia
Russula albonigra
Russula nigricans
Russula acrifolia
Russula anthracina
Russula pectinata
Russula illota
Russula subfoetens
Russula fragrantissima
Russula grata
Russula lutensis
Russula amoenolens
Russula sororia
Russula livescens
Russula insignis
Russula pectinatoides
Russula praetervisa
Russula viscida
Russula carpini
Russula cavipes
Russula aquosa
Russula laricina
Russula atropurpurea
Russula azurea
Russula zvarae
Russula minutula
Russula laeta
Russula amethystina
Russula postiana
Russula lilacea
Russula emeticicolor
Russula violacea
Russula cessans
Russula odorata
Russula gracilis
Russula solaris
Russula puellula
Russula violaceoincarnata
Russula graminea
Russula veternosa
Russula rubra
Russula persicina
Russula consobrina
Russula puellaris
Russula melitodes
Russula pseudointegra
Russula clariana
Russula integra
Russula helodes
Russula messapica
Russula versicolor
Russula badia
Russula decolorans
Russula maculata
Russula graveolens
Russula sphagnophila
Russula nitida
Russula amoenipes
Russula clavipes
Russula pascua
Russula faginea
Russula xerampelina
Russula favrei
Russula torulosa
Russula carminipes
Russula melzeri
Russula curtipes
Lactarius zonarius
Russula saliceticola
Russula melliolens
Russula caerulea
Russula claroflava
Russula vinosa
Russula dryadicola
Russula nauseosa
Russula sapinea
Russula velenovskyi
Russula aurantiaca
Russula lepidicolor
Russula paludosa
Russula cuprea
Russula brunneoviolacea
Russula pelargonia
Russula fuscorubroides
Russula atrorubens
Russula silvestris
Russula laccata
Russula nana
Russula emetica
Russula decipiens
Russula olivacea
Russula turci
Russula roseipes
Russula luteotacta
Russula betularum
Russula gracillima
Russula queletii
Russula ochroleuca
Russula alnetorum
Russula raoultii
Russula sardonia
Russula sanguinea
Russula fellea
Russula exalbicans
Russula sanguinaria
Russula foetens
Russula farinipes
Russula fragilis
Russula rosea
Russula firmula
Russula gigasperma
Russula aurea
Russula romellii
Russula rubroalba
Russula alutacea
Volvariella volvacea
Volvariella bombycina
Volvariella surrecta
Volvariella strangulata
Volvariella hypopithys
Volvariella pusilla
Volvariella caesiotincta
Volvariella taylorii
Tricholoma sulphurescens
Plicaturopsis crispa
Phyllotopsis nidulans
Hygrophoropsis aurantiaca
Hygrophoropsis rufa
Tapinella atrotomentosa
Tapinella panuoides
Chroogomphus helveticus
Chroogomphus rutilus
Chroogomphus fulmineus
Suillus cavipes
Suillus tridentinus
Suillus grevillei
Suillus bresadolae
Suillus viscidus
Suillus bovinus
Suillus plorans
Suillus granulatus
Suillus lakei
Suillus amabilis
Suillus placidus
Suillus variegatus
Suillus spraguei
Suillus americanus
Suillus flavidus
Suillus mediterraneensis
Suillus bellinii
Suillus luteus
Suillus collinitus
Gyroporus cyanescens
Gyroporus castaneus
Gyrodon lividus
Paxillus involutus
Paxillus rubicundulus
Paxillus vernalis
Paxillus ammoniavirescens
Paxillus validus
Rubinoboletus rubinus
Pseudoboletus parasiticus
Aureoboletus gentilis
Boletus speciosus
Butyriboletus fechtneri
Butyriboletus appendiculatus
Tylopilus felleus
Butyriboletus pseudoregius
Butyriboletus regius
Imperator rhodopurpureus
Imperator luteocupreus
Boletus erythropus
Strobilomyces strobilaceus
Boletus pinophilus
Boletus aereus
Tylopilus porphyrosporus
Boletus reticulatus
Leccinellum corsicum
Boletus edulis
Boletus pinetorum
Xerocomellus ripariellus
Xerocomellus dryophilus
Xerocomellus cisalpinus
Xerocomellus chrysenteron
Xanthoconium separans
Aureoboletus moravicus
Phylloporus pelletieri
Phylloporus rhodoxanthus
Xerocomus silwoodensis
Xerocomellus porosporus
Boletus subtomentosus
Boletus ferrugineus
Xerocomus chrysonemus
Leccinellum crocipodium
Leccinum duriusculum
Leccinum variicolor
Leccinum holopus
Leccinum schistophilum
Leccinum scabrum
Leccinum melaneum
Leccinum versipelle
Leccinum pulchrum
Leccinum aurantiacum
Leccinum vulpinum
Leccinum cerinum
Rhizomarasmius epidryas
Marasmius favrei
Rhodotus palmatus
Flammulina populicola
Flammulina fennae
Flammulina elastica
Flammulina velutipes
Flammulina ononidis
Hymenopellis radicata
Stropharia albonitens
Strobilurus tenacellus
Strobilurus stephanocystis
Strobilurus esculentus
Xerula pudens
Marasmius hudsonii
Marasmius epiphyllus
Rhizomarasmius undatus
Pleurotus dryinus
Pleurotus citrinopileatus
Pleurotus calyptratus
Pleurotus cornucopiae
Pleurotus pulmonarius
Cortinarius depressus
Pleurotus eryngii
Pleurotus ostreatus
Pleurotus columbinus
Hohenbuehelia unguicularis
Hohenbuehelia grisea
Hohenbuehelia culmicola
Hohenbuehelia pinacearum
Hohenbuehelia reniformis
Hohenbuehelia cyphelliformis
Hohenbuehelia mastrucata
Hohenbuehelia auriscalpium
Hohenbuehelia tremula
Hohenbuehelia petaloides
Hohenbuehelia angustata
Lepiota rufipes

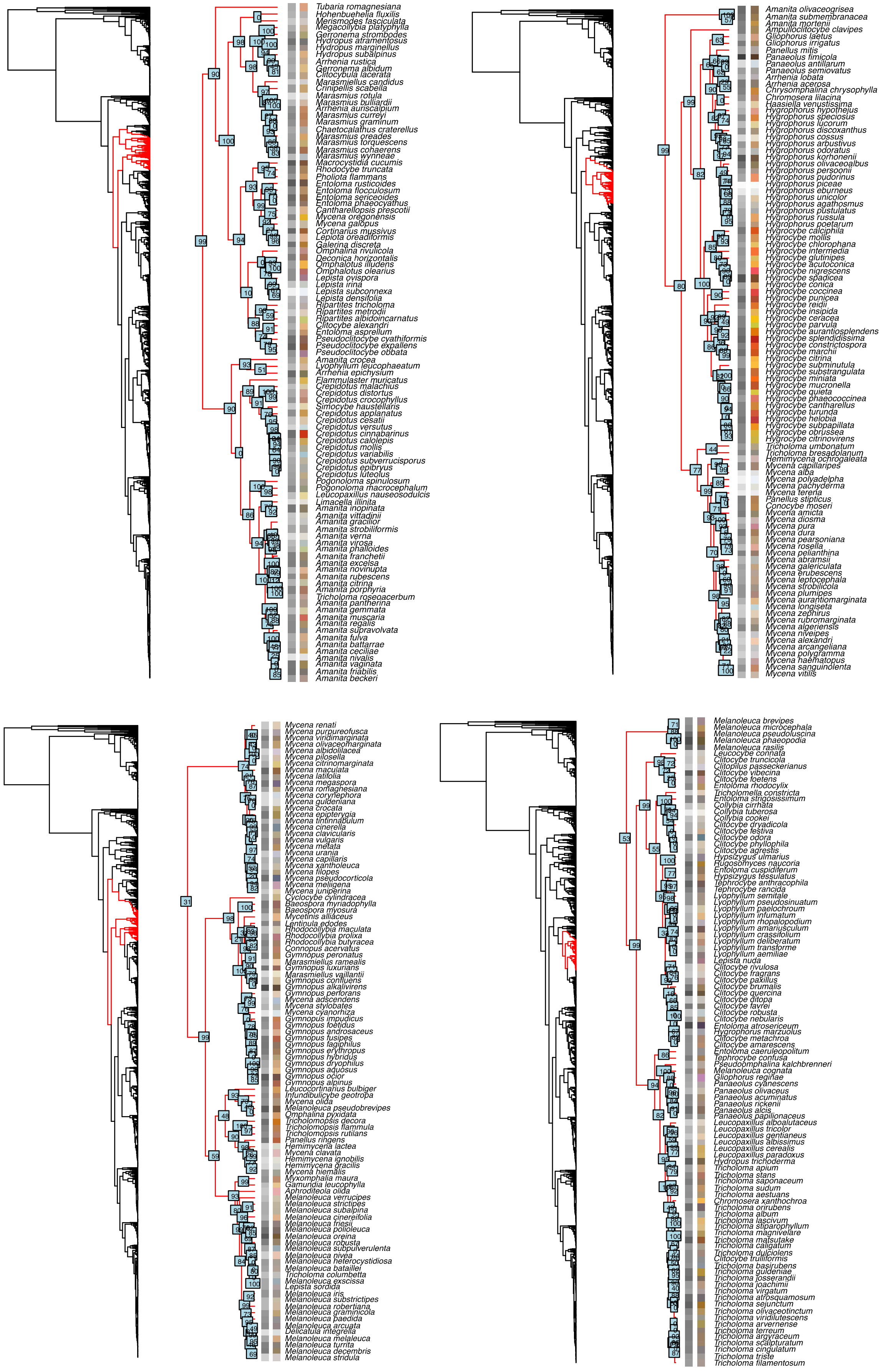
Tubaria romagnesiana
Hohenbuehelia fluxilis
Merismodes fasciculata
Megacollybia platyphylla
Gerronema strombodes
Hydropus atramentosus
Hydropus marginellus
Hydropus subalpinus
Arrhenia rustica
Gerronema albidum
Clitocybula lacerata
Marasmiellus candidus
Crinipellis scabella
Marasmius rotula
Marasmius bulliardii
Arrhenia auriscalpium
Marasmius curreyi
Marasmius graminum
Chaetocalathus craterellus
Marasmius oreades
Marasmius torquescens
Marasmius cohaerens
Marasmius wynneae
Macrocystidia cucumis
Rhodocybe truncata
Pholiota flammans
Entoloma rusticoides
Entoloma flocculosum
Entoloma sericeoides
Entoloma phaeocyathus
Cantharellopsis prescotii
Mycena oregonensis
Mycena galopus
Cortinarius mussivus
Lepiota oreadiformis
Galerina discreta
Omphalina rivulicola
Deconica horizontalis
Omphalotus illudens
Omphalotus olearius
Lepista ovispora
Lepista irina
Lepista subconnexa
Lepista densifolia
Ripartites tricholoma
Ripartites metrodii
Ripartites albidoincarnatus
Clitocybe alexandri
Entoloma asprellum
Pseudoclitocybe cyathiformis
Pseudoclitocybe expallens
Pseudoclitocybe obbata
Amanita crocea
Lyophyllum leucophaeatum
Arrhenia epichysium
Flammulaster muricatus
Crepidotus malachius
Crepidotus distortus
Crepidotus crocophyllus
Simocybe haustellaris
Crepidotus applanatus
Crepidotus cesatii
Crepidotus versutus
Crepidotus cinnabarinus
Crepidotus calolepis
Crepidotus mollis
Crepidotus variabilis
Crepidotus subverrucisporus
Crepidotus epibryus
Crepidotus luteolus
Pogonoloma spinulosum
Pogonoloma macrocephalum
Leucopaxillus nauseosodulcis
Limacella illinita
Amanita inopinata
Amanita vittadinii
Amanita gracilior
Amanita strobiliformis
Amanita verna
Amanita virosa
Amanita phalloides
Amanita franchetii
Amanita excelsa
Amanita novinupta
Amanita rubescens
Amanita citrina
Amanita porphyria
Tricholoma roseoacerbum
Amanita pantherina
Amanita gemmata
Amanita muscaria
Amanita regalis
Amanita supravolvata
Amanita fulva
Amanita battarrae
Amanita ceciliae
Amanita nivalis
Amanita vaginata
Amanita friabilis
Amanita beckeri
Amanita olivaceogrisea
Amanita submembranacea
Amanita mortenii
Ampulloclitocybe clavipes
Gliophorus laetus
Gliophorus irrigatus
Panellus mitis
Panaeolus fimicola
Panaeolus antillarum
Panaeolus semiovatus
Arrhenia lobata
Arrhenia acerosa
Chrysomphalina chrysophylla
Chromosera lilacina
Haasiella venustissima
Hygrophorus hypothejus
Hygrophorus speciosus
Hygrophorus lucorum
Hygrophorus discoxanthus
Hygrophorus cossus
Hygrophorus arbustivus
Hygrophorus odoratus
Hygrophorus korhonenii
Hygrophorus olivaceoalbus
Hygrophorus persoonii
Hygrophorus pudorinus
Hygrophorus piceae
Hygrophorus eburneus
Hygrophorus unicolor
Hygrophorus agathosmus
Hygrophorus pustulatus
Hygrophorus russula
Hygrophorus poetarum
Hygrocybe calciphila
Hygrocybe mollis
Hygrocybe chlorophana
Hygrocybe intermedia
Hygrocybe glutinipes
Hygrocybe acutoconica
Hygrocybe nigrescens
Hygrocybe spadicea
Hygrocybe conica
Hygrocybe coccinea
Hygrocybe punicea
Hygrocybe reidii
Hygrocybe insipida
Hygrocybe ceracea
Hygrocybe parvula
Hygrocybe aurantiosplendens
Hygrocybe splendidissima
Hygrocybe constrictospora
Hygrocybe marchii
Hygrocybe citrina
Hygrocybe subminutula
Hygrocybe substrangulata
Hygrocybe miniata
Hygrocybe mucronella
Hygrocybe quieta
Hygrocybe phaeococcinea
Hygrocybe cantharellus
Hygrocybe turunda
Hygrocybe helobia
Hygrocybe subpapillata
Hygrocybe obrussea
Hygrocybe citrinovirens
Tricholoma umbonatum
Tricholoma bresadolanum
Hemimycena ochrogaleata
Mycena capillaripes
Mycena alba
Mycena polyadelpha
Mycena pachyderma
Mycena terena
Panellus stipticus
Conocybe moseri
Mycena amicta
Mycena diosma
Mycena pura
Mycena dura
Mycena pearsoniana
Mycena rosella
Mycena pelianthina
Mycena abramsii
Mycena galericulata
Mycena erubescens
Mycena leptocephala
Mycena strobilicola
Mycena plumipes
Mycena aurantiomarginata
Mycena longiseta
Mycena zephirus
Mycena rubromarginata
Mycena algeriensis
Mycena niveipes
Mycena alexandri
Mycena arcangeliana
Mycena polygramma
Mycena haematopus
Mycena sanguinolenta
Mycena vitilis
Mycena renati
Mycena purpureofusca
Mycena viridimarginata
Mycena olivaceomarginata
Mycena albidolilacea
Mycena pilosella
Mycena citrinomarginata
Mycena maculata
Mycena latifolia
Mycena megaspora
Mycena romagnesiana
Mycena corynephora
Mycena guldeniana
Mycena crocata
Mycena epipterygia
Mycena tintinnabulum
Mycena cinerella
Mycena clavicularis
Mycena vulgaris
Mycena metata
Mycena urania
Mycena capillaris
Mycena xantholeuca
Mycena filopes
Mycena pseudocorticola
Mycena meliigena
Mycena juniperina
Cyclocybe cylindracea
Baeospora myriadophylla
Baeospora myosura
Mycetinis alliaceus
Lentinula edodes
Rhodocollybia maculata
Rhodocollybia prolixa
Rhodocollybia butyracea
Connopus acervatus
Gymnopus peronatus
Marasmiellus ramealis
Gymnopus luxurians
Marasmiellus vaillantii
Gymnopus confluens
Gymnopus alkalivirens
Gymnopus perforans
Mycena adscendens
Mycena stylobates
Mycena cyanorhiza
Gymnopus impudicus
Gymnopus foetidus
Gymnopus androsaceus
Gymnopus fusipes
Gymnopus fagiphilus
Gymnopus erythropus
Gymnopus hybridus
Gymnopus dryophilus
Gymnopus aquosus
Gymnopus ocior
Gymnopus alpinus
Leucocortinarius bulbiger
Infundibulicybe geotropa
Mycena olida
Melanoleuca pseudobrevipes
Omphalina pyxidata
Tricholomopsis decora
Tricholomopsis flammula
Tricholomopsis rutilans
Panellus ringens
Hemimycena lactea
Mycena clavata
Hemimycena ignobilis
Hemimycena gracilis
Mycena hiemalis
Myxomphalia maura
Gamundia leucophylla
Aphroditeola olida
Melanoleuca verrucipes
Melanoleuca strictipes
Melanoleuca subalpina
Melanoleuca cinereifolia
Melanoleuca friesii
Melanoleuca polioleuca
Melanoleuca oreina
Melanoleuca robusta
Melanoleuca subpulverulenta
Melanoleuca nivea
Melanoleuca heterocystidiosa
Melanoleuca bataillei
Tricholoma columbetta
Melanoleuca excissa
Lepista sordida
Melanoleuca iris
Melanoleuca substrictipes
Melanoleuca robertiana
Melanoleuca graminicola
Melanoleuca paedida
Melanoleuca arcuata
Delicatula integrella
Melanoleuca melaleuca
Melanoleuca turrita
Melanoleuca decembris
Melanoleuca stridula
Melanoleuca brevipes
Melanoleuca microcephala
Melanoleuca pseudoluscina
Melanoleuca phaeopodia
Melanoleuca rasilis
Leucocybe connata
Clitocybe truncicola
Clitopilus passeckerianus
Clitocybe vibecina
Clitocybe foetens
Entoloma rhodocylix
Tricholomella constricta
Entoloma strigosissimum
Collybia cirrhata
Collybia tuberosa
Collybia cookei
Clitocybe dryadicola
Clitocybe festiva
Clitocybe odora
Clitocybe phyllophila
Clitocybe agrestis
Hypsizygus ulmarius
Rugosomyces naucoria
Entoloma cuspidiferum
Hypsizygus tessulatus
Tephrocybe anthracophila
Tephrocybe rancida
Lyophyllum semitale
Lyophyllum pseudosinuatum
Lyophyllum paelochroum
Lyophyllum infumatum
Lyophyllum rhopalopodium
Lyophyllum amariusculum
Lyophyllum crassifolium
Lyophyllum deliberatum
Lyophyllum transforme
Lyophyllum aemiliae
Lepista nuda
Clitocybe rivulosa
Clitocybe fragrans
Clitocybe paxillus
Clitocybe brumalis
Clitocybe quercina
Clitocybe ditopa
Clitocybe favrei
Clitocybe robusta
Clitocybe nebularis
Entoloma atrosericeum
Hygrophorus marzuolus
Clitocybe metachroa
Clitocybe amarescens
Entoloma caeruleopolitum
Tephrocybe confusa
Pseudoomphalina kalchbrenneri
Melanoleuca cognata
Gliophorus reginae
Panaeolus cyanescens
Panaeolus olivaceus
Panaeolus acuminatus
Panaeolus rickenii
Panaeolus alcis
Panaeolus papilionaceus
Leucopaxillus alboalutaceus
Leucopaxillus tricolor
Leucopaxillus gentianeus
Leucopaxillus albissimus
Leucopaxillus cerealis
Leucopaxillus paradoxus
Hydropus trichoderma
Tricholoma apium
Tricholoma stans
Tricholoma saponaceum
Tricholoma sudum
Tricholoma aestuans
Chromosera xanthochroa
Tricholoma orirubens
Tricholoma album
Tricholoma lascivum
Tricholoma stiparophyllum
Tricholoma magnivelare
Tricholoma matsutake
Tricholoma caligatum
Tricholoma dulciolens
Clitocybe trulliformis
Tricholoma basirubens
Tricholoma guldeniae
Tricholoma josserandii
Tricholoma joachimii
Tricholoma virgatum
Tricholoma atrosquamosum
Tricholoma sejunctum
Tricholoma olivaceotinctum
Tricholoma viridilutescens
Tricholoma arvernense
Tricholoma terreum
Tricholoma argyraceum
Tricholoma scalpturatum
Tricholoma cingulatum
Tricholoma triste
Tricholoma filamentosum

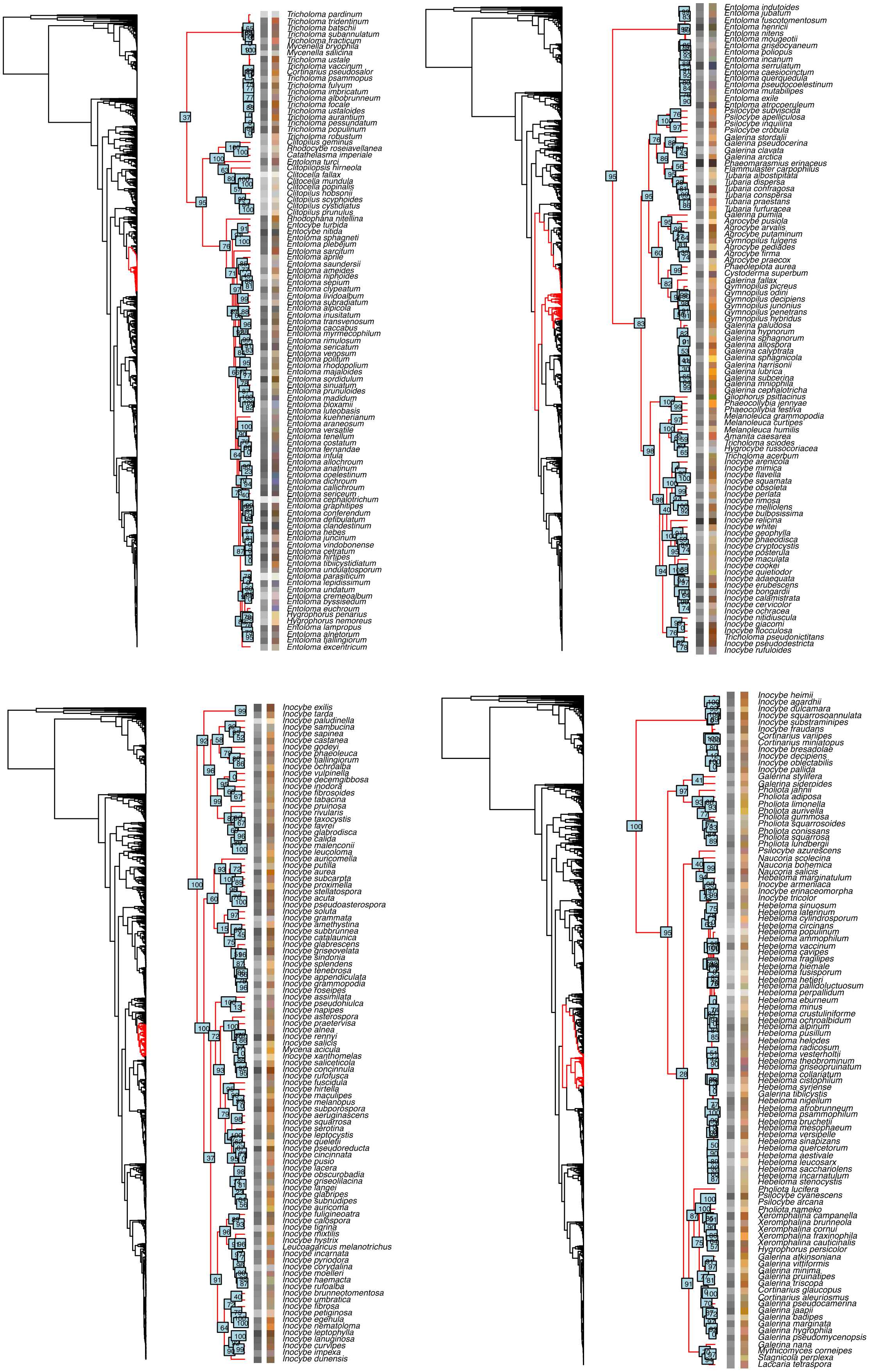
Tricholoma pardinum
Tricholoma tridentinum
Tricholoma batschii
Tricholoma subannulatum
Tricholoma fracticum
Mycenella bryophila
Mycenella salicina
Tricholoma ustale
Tricholoma vaccinum
Cortinarius pseudosalor
Tricholoma psammopus
Tricholoma fulvum
Tricholoma imbricatum
Tricholoma albobrunneum
Tricholoma focale
Tricholoma ustaloides
Tricholoma aurantium
Tricholoma pessundatum
Tricholoma populinum
Tricholoma robustum
Clitopilus geminus
Rhodocybe roseiavellanea
Catathelasma imperiale
Entoloma turci
Clitopilopsis hirneola
Clitocella fallax
Clitocella mundula
Clitocella popinalis
Clitopilus hobsonii
Clitopilus scyphoides
Clitopilus cystidiatus
Clitopilus prunulus
Rhodophana nitellina
Entocybe turbida
Entocybe nitida
Entoloma sphagneti
Entoloma plebejum
Entoloma sarcitum
Entoloma aprile
Entoloma saundersii
Entoloma ameides
Entoloma niphoides
Entoloma sepium
Entoloma clypeatum
Entoloma lividoalbum
Entoloma subradiatum
Entoloma alpicola
Entoloma inusitatum
Entoloma transvenosum
Entoloma caccabus
Entoloma myrmecophilum
Entoloma rimulosum
Entoloma sericatum
Entoloma venosum
Entoloma politum
Entoloma rhodopolium
Entoloma majaloides
Entoloma sordidulum
Entoloma sinuatum
Entoloma prunuloides
Entoloma madidum
Entoloma bloxamii
Entoloma luteobasis
Entoloma kuehnerianum
Entoloma araneosum
Entoloma versatile
Entoloma tenellum
Entoloma costatum
Entoloma fernandae
Entoloma infula
Entoloma allochroum
Entoloma anatinum
Entoloma coelestinum
Entoloma dichroum
Entoloma callichroum
Entoloma sericeum
Entoloma cephalotrichum
Entoloma graphitipes
Entoloma conferendum
Entoloma defibulatum
Entoloma clandestinum
Entoloma hebes
Entoloma juncinum
Entoloma vindobonense
Entoloma cetratum
Entoloma hirtipes
Entoloma tibiicystidiatum
Entoloma undulatosporum
Entoloma parasiticum
Entoloma lepidissimum
Entoloma undatum
Entoloma cremeoalbum
Entoloma byssisedum
Entoloma euchroum
Hygrophorus penarius
Hygrophorus nemoreus
Entoloma lampropus
Entoloma alnetorum
Entoloma tjallingiorum
Entoloma excentricum
Entoloma indutoides
Entoloma jubatum
Entoloma fuscotomentosum
Entoloma henricii
Entoloma nitens
Entoloma mougeotii
Entoloma griseocyaneum
Entoloma poliopus
Entoloma incanum
Entoloma serrulatum
Entoloma caesiocinctum
Entoloma querquedula
Entoloma pseudocoelestinum
Entoloma mutabilipes
Entoloma exile
Entoloma atrocoeruleum
Psilocybe subviscida
Psilocybe apelliculosa
Psilocybe inquilina
Psilocybe crobula
Galerina stordalii
Galerina pseudocerina
Galerina clavata
Galerina arctica
Phaeomarasmius erinaceus
Flammulaster carpophilus
Tubaria albostipitata
Tubaria dispersa
Tubaria confragosa
Tubaria conspersa
Tubaria praestans
Tubaria furfuracea
Galerina pumila
Agrocybe pusiola
Agrocybe arvalis
Agrocybe putaminum
Gymnopilus fulgens
Agrocybe pediades
Agrocybe firma
Agrocybe praecox
Phaeolepiota aurea
Cystoderma superbum
Galerina fallax
Gymnopilus picreus
Gymnopilus odini
Gymnopilus decipiens
Gymnopilus junonius
Gymnopilus penetrans
Gymnopilus hybridus
Galerina paludosa
Galerina hypnorum
Galerina sphagnorum
Galerina allospora
Galerina calyptrata
Galerina sphagnicola
Galerina harrisonii
Galerina lubrica
Galerina subcerina
Galerina mniophila
Galerina cephalotricha
Gliophorus psittacinus
Phaeocollybia jennyae
Phaeocollybia festiva
Melanoleuca grammopodia
Melanoleuca curtipes
Melanoleuca humilis
Amanita caesarea
Tricholoma sciodes
Hygrocybe russocoriacea
Tricholoma acerbum
Inocybe arenicola
Inocybe mimica
Inocybe flavella
Inocybe squamata
Inocybe obsoleta
Inocybe perlata
Inocybe rimosa
Inocybe melliolens
Inocybe bulbosissima
Inocybe relicina
Inocybe whitei
Inocybe geophylla
Inocybe phaeodisca
Inocybe cryptocystis
Inocybe posterula
Inocybe maculata
Inocybe cookei
Inocybe quietiodor
Inocybe adaequata
Inocybe erubescens
Inocybe bongardii
Inocybe calamistrata
Inocybe cervicolor
Inocybe ochracea
Inocybe nitidiuscula
Inocybe giacomi
Inocybe flocculosa
Tricholoma pseudonictitans
Inocybe pseudodestricta
Inocybe rufuloides
Inocybe exilis
Inocybe tarda
Inocybe paludinella
Inocybe sambucina
Inocybe sapinea
Inocybe castanea
Inocybe godeyi
Inocybe phaeoleuca
Inocybe tjallingiorum
Inocybe ochroalba
Inocybe vulpinella
Inocybe decemgibbosa
Inocybe inodora
Inocybe fibrosoides
Inocybe tabacina
Inocybe pruinosa
Inocybe rivularis
Inocybe taxocystis
Inocybe favrei
Inocybe glabrodisca
Inocybe calida
Inocybe malenconii
Inocybe leucoloma
Inocybe auricomella
Inocybe putilla
Inocybe aurea
Inocybe subcarpta
Inocybe proximella
Inocybe stellatospora
Inocybe acuta
Inocybe pseudoasterospora
Inocybe soluta
Inocybe grammata
Inocybe amethystina
Inocybe subbrunnea
Inocybe catalaunica
Inocybe glabrescens
Inocybe griseovelata
Inocybe sindonia
Inocybe splendens
Inocybe tenebrosa
Inocybe appendiculata
Inocybe grammopodia
Inocybe roseipes
Inocybe assimilata
Inocybe pseudohiulca
Inocybe napipes
Inocybe asterospora
Inocybe praetervisa
Inocybe alnea
Inocybe rennyi
Inocybe salicis
Mycena acicula
Inocybe xanthomelas
Inocybe saliceticola
Inocybe concinnula
Inocybe rufofusca
Inocybe fuscidula
Inocybe hirtella
Inocybe maculipes
Inocybe melanopus
Inocybe subporospora
Inocybe aeruginascens
Inocybe squarrosa
Inocybe serotina
Inocybe leptocystis
Inocybe queletii
Inocybe pseudoreducta
Inocybe cincinnata
Inocybe pusio
Inocybe lacera
Inocybe obscurobadia
Inocybe griseolilacina
Inocybe langei
Inocybe glabripes
Inocybe subnudipes
Inocybe auricoma
Inocybe fuligineoatra
Inocybe calospora
Inocybe tigrina
Inocybe mixtilis
Inocybe hystrix
Leucoagaricus melanotrichus
Inocybe incarnata
Inocybe pyriodora
Inocybe corydalina
Inocybe moelleri
Inocybe haemacta
Inocybe rufoalba
Inocybe brunneotomentosa
Inocybe umbratica
Inocybe fibrosa
Inocybe petiginosa
Inocybe egenula
Inocybe nematoloma
Inocybe leptophylla
Inocybe lanuginosa
Inocybe curvipes
Inocybe impexa
Inocybe dunensis
Inocybe heimii
Inocybe agardhii
Inocybe dulcamara
Inocybe squarrosoannulata
Inocybe substraminipes
Inocybe fraudans
Cortinarius variipes
Cortinarius miniatopus
Inocybe bresadolae
Inocybe decipiens
Inocybe oblectabilis
Inocybe pallida
Galerina stylifera
Galerina sideroides
Pholiota jahnii
Pholiota adiposa
Pholiota limonella
Pholiota aurivella
Pholiota gummosa
Pholiota squarrosoides
Pholiota conissans
Pholiota squarrosa
Pholiota lundbergii
Psilocybe azurescens
Naucoria scolecina
Naucoria bohemica
Naucoria salicis
Hebeloma marginatulum
Inocybe armeniaca
Inocybe erinaceomorpha
Inocybe tricolor
Hebeloma sinuosum
Hebeloma laterinum
Hebeloma cylindrosporum
Hebeloma circinans
Hebeloma populinum
Hebeloma ammophilum
Hebeloma vaccinum
Hebeloma cavipes
Hebeloma fragilipes
Hebeloma hiemale
Hebeloma fusisporum
Hebeloma hetieri
Hebeloma pallidoluctuosum
Hebeloma perpallidum
Hebeloma eburneum
Hebeloma minus
Hebeloma crustuliniforme
Hebeloma ochroalbidum
Hebeloma alpinum
Hebeloma pusillum
Hebeloma helodes
Hebeloma radicosum
Hebeloma vesterholtii
Hebeloma theobrominum
Hebeloma griseopruinatum
Hebeloma collariatum
Hebeloma cistophilum
Hebeloma syrjense
Galerina tibiicystis
Hebeloma nigellum
Hebeloma atrobrunneum
Hebeloma psammophilum
Hebeloma bruchetii
Hebeloma mesophaeum
Hebeloma versipelle
Hebeloma sinapizans
Hebeloma quercetorum
Hebeloma aestivale
Hebeloma leucosarx
Hebeloma sacchariolens
Hebeloma incarnatulum
Hebeloma stenocystis
Pholiota lucifera
Psilocybe cyanescens
Psilocybe arcana
Pholiota nameko
Xeromphalina campanella
Xeromphalina brunneola
Xeromphalina cornui
Xeromphalina fraxinophila
Xeromphalina cauticinalis
Hygrophorus persicolor
Galerina atkinsoniana
Galerina vittiformis
Galerina minima
Galerina pruinatipes
Galerina triscopa
Cortinarius glaucopus
Cortinarius aleuriosmus
Galerina pseudocamerina
Galerina jaapii
Galerina badipes
Galerina marginata
Galerina hygrophila
Galerina pseudomycenopsis
Galerina nana
Mythicomyces corneipes
Stagnicola perplexa
Laccaria tetraspora

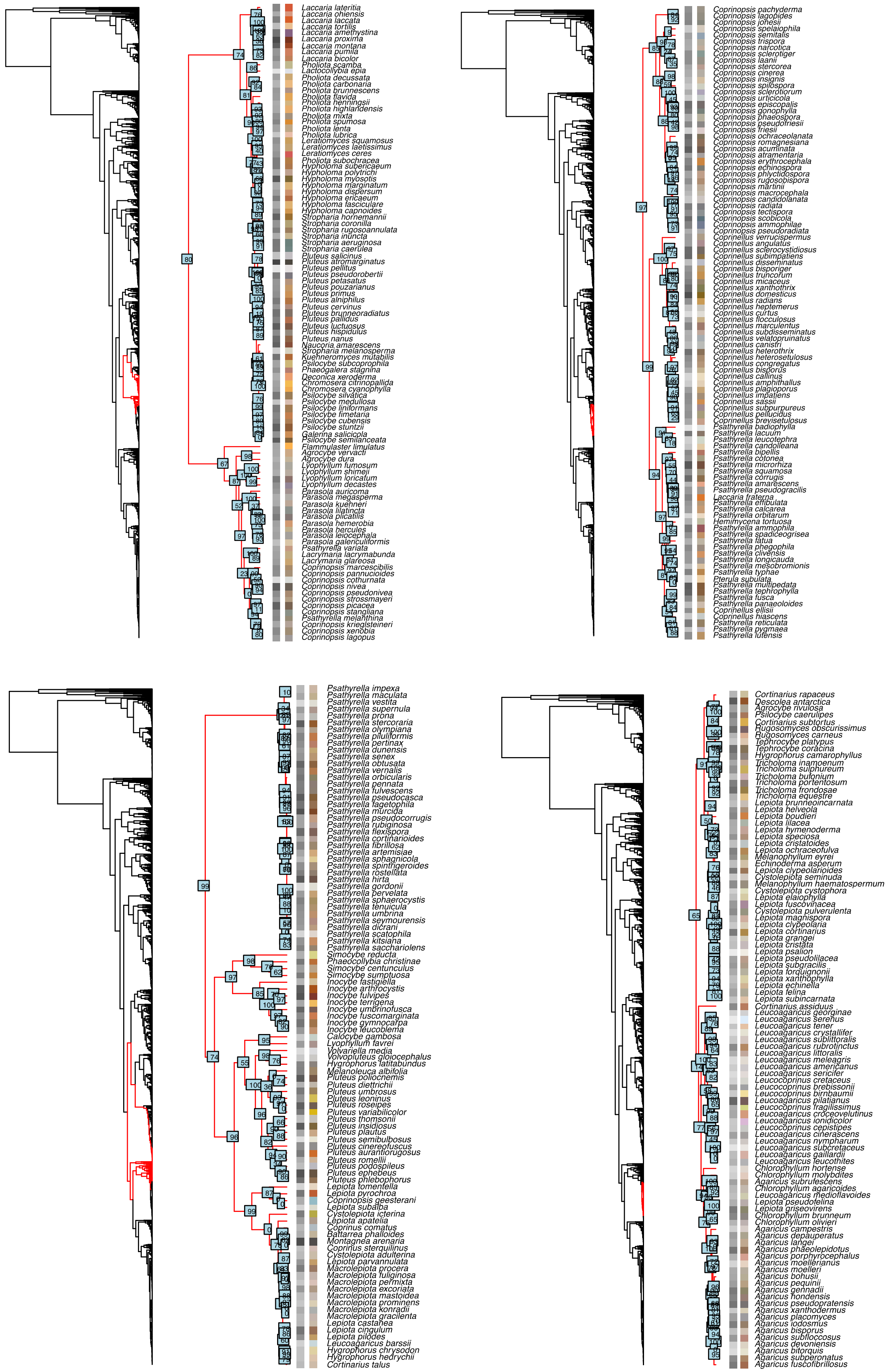
Laccaria laterita
Laccaria ohiensis
Laccaria laccata
Laccaria tortilis
Laccaria amethystina
Laccaria proxima
Laccaria montana
Laccaria pumila
Laccaria bicolor
Pholiota scamba
Lactocollybia epia
Pholiota decussata
Pholiota carbonaria
Pholiota brunnescens
Pholiota flavida
Pholiota henningsii
Pholiota highlandensis
Pholiota mixta
Pholiota spumosa
Pholiota lenta
Pholiota lubrica
Leratiomyces squamosus
Leratiomyces laetissimus
Leratiomyces ceres
Pholiota subochracea
Hypholoma subericaeum
Hypholoma polytrichi
Hypholoma myosotis
Hypholoma marginatum
Hypholoma dispersum
Hypholoma ericaeum
Hypholoma fasciculare
Hypholoma capnoides
Stropharia hornemannii
Stropharia coronilla
Stropharia rugosoannulata
Stropharia inuncta
Stropharia aeruginosa
Stropharia caerulea
Pluteus salicinus
Pluteus atromarginatus
Pluteus pellitus
Pluteus pseudorobertii
Pluteus petasatus
Pluteus pouzarianus
Pluteus primus
Pluteus alniphilus
Pluteus cervinus
Pluteus brunneoradiatus
Pluteus pallidus
Pluteus luctuosus
Pluteus hispidulus
Pluteus nanus
Naucoria amarescens
Stropharia melanosperma
Kuehneromyces mutabilis
Psilocybe subcoprophila
Phaeogalera stagnina
Deconica xeroderma
Chromosera citrinopallida
Chromosera cyanophylla
Psilocybe silvatica
Psilocybe medullosa
Psilocybe liniformans
Psilocybe fimetaria
Psilocybe cubensis
Psilocybe stuntzii
Galerina salicicola
Psilocybe semilanceata
Flammulaster limulatus
Agrocybe vervacti
Agrocybe dura
Lyophyllum fumosum
Lyophyllum shimeji
Lyophyllum loricatum
Lyophyllum decastes
Parasola auricoma
Parasola megasperma
Parasola kuehneri
Parasola lilatincta
Parasola plicatilis
Parasola hemerobia
Parasola hercules
Parasola leiocephala
Parasola galericuliformis
Psathyrella variata
Lacrymaria lacrymabunda
Lacrymaria glareosa
Coprinopsis marcescibilis
Coprinopsis pannucioides
Coprinopsis cothurnata
Coprinopsis nivea
Coprinopsis pseudonivea
Coprinopsis strossmayeri
Coprinopsis picacea
Coprinopsis stangliana
Psathyrella melanthina
Coprinopsis kriegsteineri
Coprinopsis xenobia
Coprinopsis lagopus
Coprinopsis pachyderma
Coprinopsis lagopides
Coprinopsis jonesii
Coprinopsis spelaiophila
Coprinopsis semitalis
Coprinopsis trispora
Coprinopsis narcotica
Coprinopsis sclerotiger
Coprinopsis laanii
Coprinopsis stercorea
Coprinopsis cinerea
Coprinopsis insignis
Coprinopsis spilospora
Coprinopsis sclerotiorum
Coprinopsis urticicola
Coprinopsis episcopalis
Coprinopsis gonophylla
Coprinopsis phaeospora
Coprinopsis pseudofriesii
Coprinopsis friesii
Coprinopsis ochraceolanata
Coprinopsis romagnesiana
Coprinopsis acuminata
Coprinopsis atramentaria
Coprinopsis erythrocephala
Coprinopsis echinospora
Coprinopsis phlyctidospora
Coprinopsis rugosobispora
Coprinopsis martinii
Coprinopsis macrocephala
Coprinopsis candidolanata
Coprinopsis radiata
Coprinopsis tectispora
Coprinopsis scobicola
Coprinopsis ammophilae
Coprinopsis pseudoradiata
Coprinellus verrucispermus
Coprinellus angulatus
Coprinellus sclerocystidiosus
Coprinellus subimpatiens
Coprinellus disseminatus
Coprinellus bisporiger
Coprinellus truncorum
Coprinellus micaceus
Coprinellus xanthothrix
Coprinellus domesticus
Coprinellus radians
Coprinellus heptemerus
Coprinellus curtus
Coprinellus flocculosus
Coprinellus marculentus
Coprinellus subdisseminatus
Coprinellus velatopruinatus
Coprinellus canistri
Coprinellus heterothrix
Coprinellus heterosetulosus
Coprinellus congregatus
Coprinellus bisporus
Coprinellus callinus
Coprinellus amphithallus
Coprinellus plagioporus
Coprinellus impatiens
Coprinellus sassii
Coprinellus subpurpureus
Coprinellus pellucidus
Coprinellus brevisetulosus
Psathyrella badiophylla
Psathyrella lacuum
Psathyrella leucotephra
Psathyrella candolleana
Psathyrella bipellis
Psathyrella cotonea
Psathyrella microrhiza
Psathyrella squamosa
Psathyrella corrugis
Psathyrella amarescens
Psathyrella pseudogracilis
Laccaria fraterna
Psathyrella effibulata
Psathyrella calcarea
Psathyrella orbitarum
Hemimycena tortuosa
Psathyrella ammophila
Psathyrella spadiceogrisea
Psathyrella fatua
Psathyrella phegophila
Psathyrella clivensis
Psathyrella longicauda
Psathyrella mesobromionis
Psathyrella typhae
Pterula subulata
Psathyrella multipedata
Psathyrella tephrophylla
Psathyrella fusca
Psathyrella panaeoloides
Coprinellus ellisii
Coprinellus hiascens
Psathyrella reticulata
Psathyrella pygmaea
Psathyrella lutensis
Psathyrella impexa
Psathyrella maculata
Psathyrella vestita
Psathyrella supernula
Psathyrella prona
Psathyrella stercoraria
Psathyrella olympiana
Psathyrella piluliformis
Psathyrella pertinax
Psathyrella dunensis
Psathyrella senex
Psathyrella obtusata
Psathyrella vernalis
Psathyrella orbicularis
Psathyrella pennata
Psathyrella fulvescens
Psathyrella pseudocasca
Psathyrella fagetophila
Psathyrella murcida
Psathyrella pseudocorrugis
Psathyrella rubiginosa
Psathyrella flexispora
Psathyrella cortinarioides
Psathyrella fibrillosa
Psathyrella artemisiae
Psathyrella sphagnicola
Psathyrella spintrigeroides
Psathyrella rostellata
Psathyrella hirta
Psathyrella gordonii
Psathyrella pervelata
Psathyrella sphaerocystis
Psathyrella tenuicula
Psathyrella umbrina
Psathyrella seymourensis
Psathyrella dicrani
Psathyrella scatophila
Psathyrella kitsiana
Psathyrella saccharolens
Simocybe reducta
Phaeocollybia christinae
Simocybe centunculus
Simocybe sumptuosa
Inocybe fastigiella
Inocybe arthrocystis
Inocybe fulvipes
Inocybe terrigena
Inocybe umbrinofusca
Inocybe fuscomarginata
Inocybe gymnocarpa
Inocybe leucoblema
Calocybe gambosa
Lyophyllum favrei
Volvariella media
Volvopluteus gloiocephalus
Hygrophorus latitabundus
Melanoleuca albifolia
Pluteus poliocnemis
Pluteus diettrichii
Pluteus umbrosus
Pluteus leoninus
Pluteus roseipes
Pluteus variabilicolor
Pluteus thomsonii
Pluteus insidiosus
Pluteus plautus
Pluteus semibulbosus
Pluteus cinereofuscus
Pluteus aurantiorugosus
Pluteus romellii
Pluteus podospileus
Pluteus ephebeus
Pluteus phlebophorus
Lepiota tomentella
Lepiota pyrochroa
Coprinopsis geesterani
Lepiota subalba
Cystolepiota icterina
Lepiota apatelia
Coprinus comatus
Battarrea phalloides
Montagnea arenaria
Coprinus sterquilinus
Cystolepiota adulterina
Lepiota parvannulata
Macrolepiota procera
Macrolepiota fuliginosa
Macrolepiota permixta
Macrolepiota excoriata
Macrolepiota mastoidea
Macrolepiota prominens
Macrolepiota konradii
Macrolepiota gracilenta
Lepiota castanea
Lepiota cingulum
Lepiota pilodes
Leucoagaricus barssii
Hygrophorus chrysodon
Hygrophorus hedrychii
Cortinarius talus
Cortinarius rapaceus
Descolea antarctica
Agrocybe rivulosa
Psilocybe caerulipes
Cortinarius subtortus
Rugosomyces obscurissimus
Rugosomyces carneus
Tephrocybe platypus
Tephrocybe coracina
Hygrophorus camarophyllus
Tricholoma inamoenum
Tricholoma sulphureum
Tricholoma bufonium
Tricholoma portentosum
Tricholoma frondosae
Tricholoma equestre
Lepiota brunneoincarnata
Lepiota helveola
Lepiota boudieri
Lepiota lilacea
Lepiota hymenoderma
Lepiota speciosa
Lepiota cristatoides
Lepiota ochraceofulva
Melanophyllum eyrei
Echinoderma asperum
Lepiota clypeolarioides
Cystolepiota seminuda
Melanophyllum haematospermum
Cystolepiota cystophora
Lepiota elaiophylla
Lepiota fuscovinacea
Cystolepiota pulverulenta
Lepiota magnispora
Lepiota clypeolaria
Lepiota cortinarius
Lepiota grangei
Lepiota cristata
Lepiota psalion
Lepiota pseudolilacea
Lepiota subgracilis
Lepiota forquignonii
Lepiota xanthophylla
Lepiota echinella
Lepiota felina
Lepiota subincarnata
Cortinarius assiduus
Leucoagaricus georginae
Leucoagaricus serenus
Leucoagaricus tener
Leucoagaricus crystallifer
Leucoagaricus sublittoralis
Leucoagaricus rubrotinctus
Leucoagaricus littoralis
Leucoagaricus meleagris
Leucoagaricus americanus
Leucoagaricus sericifer
Leucocoprinus cretaceus
Leucocoprinus brebissonii
Leucocoprinus birnbaumii
Leucoagaricus pilatianus
Leucocoprinus fragilissimus
Leucoagaricus croceovelutinus
Leucoagaricus ionidicolor
Leucocoprinus cepistipes
Leucoagaricus cinerascens
Leucoagaricus nympharum
Leucoagaricus subcretaceus
Leucoagaricus gaillardii
Leucoagaricus leucothites
Chlorophyllum hortense
Chlorophyllum molybdites
Agaricus subrufescens
Chlorophyllum agaricoides
Leucoagaricus medioflavoides
Lepiota pseudofelina
Lepiota griseovirens
Chlorophyllum brunneum
Chlorophyllum olivieri
Agaricus campestris
Agaricus depauperatus
Agaricus langei
Agaricus phaeolepidotus
Agaricus porphyrocephalus
Agaricus moellerianus
Agaricus moelleri
Agaricus bohusii
Agaricus pequinii
Agaricus gennadii
Agaricus hondensis
Agaricus pseudopratensis
Agaricus xanthodermus
Agaricus placomyces
Agaricus iodosmus
Agaricus bisporus
Agaricus subfloccosus
Agaricus devoniensis
Agaricus bitorquis
Agaricus subperonatus
Agaricus fuscofibrillosus

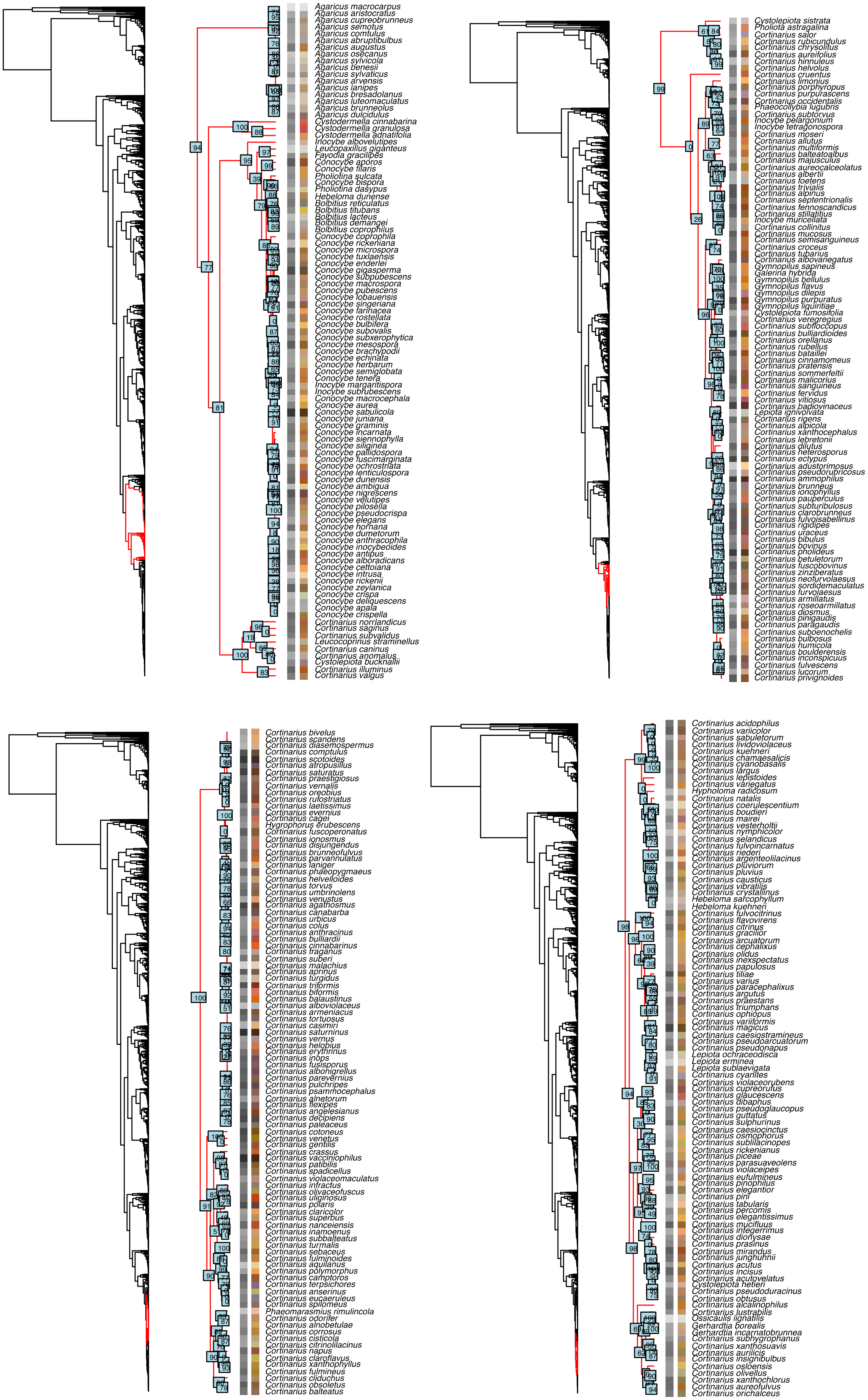
Agaricus macrocarpus
Agaricus aristocratus
Agaricus cupreobrunneus
Agaricus semotus
Agaricus comtulus
Agaricus abruptibulbus
Agaricus augustus
Agaricus osecanus
Agaricus sylvicola
Agaricus benesii
Agaricus sylvaticus
Agaricus arvensis
Agaricus lanipes
Agaricus bresadolanus
Agaricus luteomaculatus
Agaricus brunneolus
Agaricus dulcidulus
Cystodermella cinnabarina
Cystodermella granulosa
Cystodermella adnatifolia
Inocybe albovelutipes
Leucopaxillus giganteus
Fayodia gracilipes
Conocybe aporos
Conocybe filaris
Pholiotina sulcata
Conocybe bispora
Pholiotina dasypus
Hebeloma dunense
Bolbitius reticulatus
Bolbitius titubans
Bolbitius lacteus
Bolbitius demangei
Bolbitius coprophilus
Conocybe coprophila
Conocybe rickeniana
Conocybe microspora
Conocybe tuxlaensis
Conocybe enderlei
Conocybe gigasperma
Conocybe subpubescens
Conocybe macrospora
Conocybe pubescens
Conocybe lobauensis
Conocybe singeriana
Conocybe farinacea
Conocybe rostellata
Conocybe bulbifera
Conocybe subovalis
Conocybe subxerophytica
Conocybe mesospora
Conocybe brachypodii
Conocybe echinata
Conocybe herbarum
Conocybe semiglobata
Conocybe tenera
Inocybe margaritispora
Inocybe subrubescens
Conocybe macrocephala
Conocybe aurea
Conocybe sabulicola
Conocybe juniana
Conocybe graminis
Conocybe incarnata
Conocybe siennophylla
Conocybe siliginea
Conocybe pallidospora
Conocybe fuscimarginata
Conocybe ochrostriata
Conocybe lenticulospora
Conocybe dunensis
Conocybe ambigua
Conocybe nigrescens
Conocybe velutipes
Conocybe pilosella
Conocybe pseudocrispa
Conocybe elegans
Conocybe hornana
Conocybe dumetorum
Conocybe anthracophila
Conocybe inocybeoides
Conocybe antipus
Conocybe alboradicans
Conocybe cettoiana
Conocybe intrusa
Conocybe rickenii
Conocybe zeylanica
Conocybe crispa
Conocybe deliquescens
Conocybe apala
Conocybe crispella
Cortinarius norrlandicus
Cortinarius saginus
Cortinarius subvalidus
Leucocoprinus straminellus
Cortinarius caninus
Cortinarius anomalus
Cystolepiota bucknallii
Cortinarius illuminus
Cortinarius valgus
Cystolepiota sistrata
Pholiota astragalina
Cortinarius salor
Cortinarius rubicundulus
Cortinarius chrysolitus
Cortinarius aureifolius
Cortinarius hinnuleus
Cortinarius helvolus
Cortinarius cruentus
Cortinarius limonius
Cortinarius porphyropus
Cortinarius purpurascens
Cortinarius occidentalis
Phaeocollybia lugubris
Cortinarius subtorvus
Inocybe pelargonium
Inocybe tetragonospora
Cortinarius moseri
Cortinarius allutus
Cortinarius multiformis
Cortinarius balteatoalbus
Cortinarius majusculus
Cortinarius aureocalceolatus
Cortinarius albertii
Cortinarius foetens
Cortinarius trivialis
Cortinarius alpinus
Cortinarius septentrionalis
Cortinarius fennoscandicus
Cortinarius stillatitius
Inocybe muricellata
Cortinarius collinitus
Cortinarius mucosus
Cortinarius semisanguineus
Cortinarius croceus
Cortinarius tubarius
Cortinarius albovariegatus
Gymnopilus sapineus
Galerina hybrida
Gymnopilus bellulus
Gymnopilus flavus
Gymnopilus dilepis
Gymnopilus purpuratus
Gymnopilus liquiritiae
Cystolepiota fumosifolia
Cortinarius veregregius
Cortinarius subfloccopus
Cortinarius bulliardioides
Cortinarius orellanus
Cortinarius rubellus
Cortinarius bataillei
Cortinarius cinnamomeus
Cortinarius pratensis
Cortinarius sommerfeltii
Cortinarius malicorius
Cortinarius sanguineus
Cortinarius fervidus
Cortinarius vitiosus
Cortinarius badiovinaceus
Lepiota ignivolvata
Cortinarius rigens
Cortinarius alpicola
Cortinarius xanthocephalus
Cortinarius lebretonii
Cortinarius dilutus
Cortinarius heterosporus
Cortinarius ectypus
Cortinarius adustorimosus
Cortinarius pseudorubricosus
Cortinarius ammophilus
Cortinarius brunneus
Cortinarius ionophyllus
Cortinarius paupercuius
Cortinarius subturibulosus
Cortinarius clarobrunneus
Cortinarius fulvoisabellinus
Cortinarius rigidipes
Cortinarius uraceus
Cortinarius bibulus
Cortinarius bovinus
Cortinarius pholideus
Cortinarius betuletorum
Cortinarius fuscobovinus
Cortinarius zinziberatus
Cortinarius neofurvolaesus
Cortinarius sordidemaculatus
Cortinarius furvolaesus
Cortinarius armillatus
Cortinarius roseoarmillatus
Cortinarius diosmus
Cortinarius pinigaudis
Cortinarius paragaudis
Cortinarius suboenochelis
Cortinarius bulbosus
Cortinarius humicola
Cortinarius boulderensis
Cortinarius inconspicuus
Cortinarius fulvescens
Cortinarius lucorum
Cortinarius privignoides
Cortinarius bivelus
Cortinarius scandens
Cortinarius diasemospermus
Cortinarius comptulus
Cortinarius scotoides
Cortinarius atropusillus
Cortinarius saturatus
Cortinarius praestigiosus
Cortinarius vernalis
Cortinarius oreobius
Cortinarius rufostriatus
Cortinarius laetissimus
Cortinarius evernius
Cortinarius cagei
Hygrophorus erubescens
Cortinarius fuscoperonatus
Cortinarius ignosmus
Cortinarius disjungendus
Cortinarius brunneofulvus
Cortinarius parvannulatus
Cortinarius laniger
Cortinarius phaeopygmaeus
Cortinarius helvelloides
Cortinarius torvus
Cortinarius umbrinolens
Cortinarius venustus
Cortinarius agathosmus
Cortinarius canabarba
Cortinarius urbicus
Cortinarius colus
Cortinarius anthracinus
Cortinarius bulliardii
Cortinarius cinnabarinus
Cortinarius traganus
Cortinarius suberi
Cortinarius malachius
Cortinarius aprinus
Cortinarius turgidus
Cortinarius triformis
Cortinarius biformis
Cortinarius balaustinus
Cortinarius alboviolaceus
Cortinarius armeniacus
Cortinarius tortuosus
Cortinarius casimiri
Cortinarius saturninus
Cortinarius vernus
Cortinarius helobius
Cortinarius erythrinus
Cortinarius inops
Cortinarius fusisporus
Cortinarius albonigrellus
Cortinarius parevernius
Cortinarius pulchripes
Cortinarius psammocephalus
Cortinarius alnetorum
Cortinarius flexipes
Cortinarius angelesianus
Cortinarius decipiens
Cortinarius paleaceus
Cortinarius cotoneus
Cortinarius venetus
Cortinarius gentilis
Cortinarius crassus
Cortinarius vaccinophilus
Cortinarius patibilis
Cortinarius spadicellus
Cortinarius violaceomaculatus
Cortinarius infractus
Cortinarius olivaceofuscus
Cortinarius uliginosus
Cortinarius polaris
Cortinarius claricolor
Cortinarius superbus
Cortinarius nanceiensis
Cortinarius inamoenus
Cortinarius subbalteatus
Cortinarius turmalis
Cortinarius sebaceus
Cortinarius fulminoides
Cortinarius aquilanus
Cortinarius polymorphus
Cortinarius camptoros
Cortinarius terpsichores
Cortinarius anserinus
Cortinarius eucaeruleus
Cortinarius spilomeus
Phaeomarasmius rimulincola
Cortinarius odorifer
Cortinarius alnobetulae
Cortinarius corrosus
Cortinarius citrinolilacinus
Cortinarius napus
Cortinarius claroflavus
Cortinarius xanthophyllus
Cortinarius fulmineus
Cortinarius cliduchus
Cortinarius obsoletus
Cortinarius balteatus
Cortinarius acidophilus
Cortinarius variicolor
Cortinarius sabuletorum
Cortinarius lividoviolaceus
Cortinarius kuehneri
Cortinarius chamaesalicis
Cortinarius cyanobasalis
Cortinarius largus
Cortinarius lepistoides
Cortinarius variegatus
Hypholoma radicosum
Cortinarius natalis
Cortinarius coerulescentium
Cortinarius boudieri
Cortinarius mairei
Cortinarius vesterholtii
Cortinarius nymphicolor
Cortinarius selandicus
Cortinarius fulvoincarnatus
Cortinarius riederi
Cortinarius argenteolilacinus
Cortinarius pluviorum
Cortinarius pluvius
Cortinarius causticus
Cortinarius vibratilis
Cortinarius crystallinus
Hebeloma sarcophyllum
Hebeloma kuehneri
Cortinarius fulvocitrinus
Cortinarius flavovirens
Cortinarius citrinus
Cortinarius gracilior
Cortinarius arcuatorum
Cortinarius cephalixus
Cortinarius olidus
Cortinarius inexspectatus
Cortinarius papulosus
Cortinarius tiliae
Cortinarius varius
Cortinarius paracephalixus
Cortinarius argutus
Cortinarius praestans
Cortinarius triumphans
Cortinarius ophiopus
Cortinarius variiformis
Cortinarius magicus
Cortinarius caesiostramineus
Cortinarius pseudoarcuatorum
Cortinarius pseudonapus
Lepiota ochraceodisca
Lepiota erminea
Lepiota sublaevigata
Cortinarius cyanites
Cortinarius violaceorubens
Cortinarius cupreorufus
Cortinarius glaucescens
Cortinarius dibaphus
Cortinarius pseudoglaucopus
Cortinarius guttatus
Cortinarius sulphurinus
Cortinarius caesiocinctus
Cortinarius osmophorus
Cortinarius sublilacinopes
Cortinarius rickenianus
Cortinarius piceae
Cortinarius parasuaveolens
Cortinarius violaceipes
Cortinarius eufulmineus
Cortinarius pinophilus
Cortinarius elegantior
Cortinarius pini
Cortinarius tabularis
Cortinarius percomis
Cortinarius elegantissimus
Cortinarius mucifluus
Cortinarius integerrimus
Cortinarius dionysae
Cortinarius prasinus
Cortinarius mirandus
Cortinarius junghuhnii
Cortinarius acutus
Cortinarius incisus
Cortinarius acutovelatus
Cystolepiota hetieri
Cortinarius pseudoduracinus
Cortinarius obtusus
Cortinarius alcalinophilus
Cortinarius lustrabilis
Ossicaulis lignatilis
Gerhardtia borealis
Gerhardtia incarnatobrunnea
Cortinarius subhygrophanus
Cortinarius xanthosuavis
Cortinarius aurilicis
Cortinarius insignibulbus
Cortinarius osloensis
Cortinarius olivellus
Cortinarius xanthochlorus
Cortinarius aureofulvus
Cortinarius orichalceus

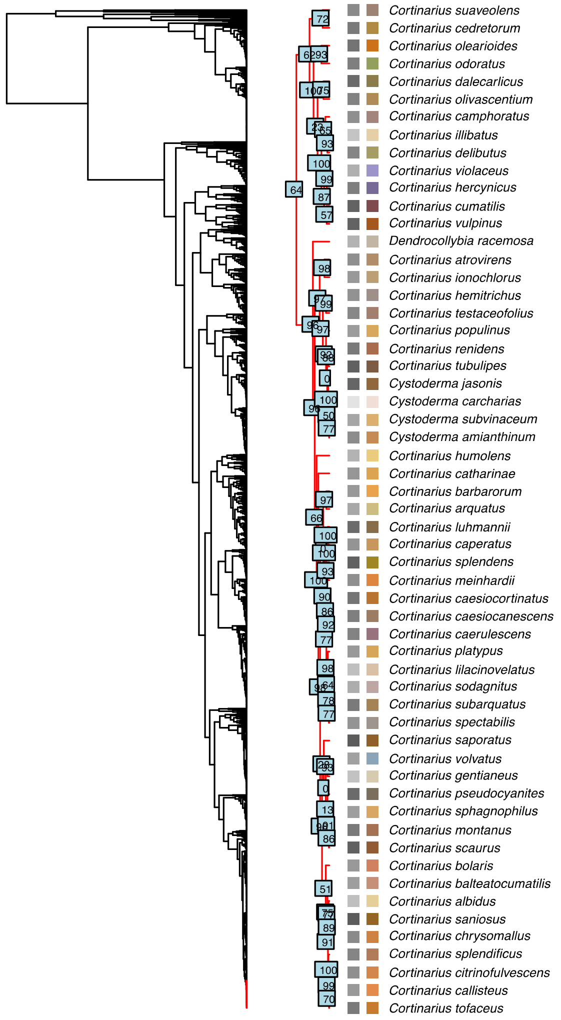

Supplementary Figure 3. Maximum-likelihood phylogeny. Branch support is given as Shimodaira–Hasegawa approximate likelihood ratio tests (SH-aLRT). The phylogeny was produced based on a mega-phylogeny approach (see ‘Mega-phylogeny approach’ in Methods). Averaged lightness and hue values are provided with the tip labels. Source data are provided in Supplementary Data 2.

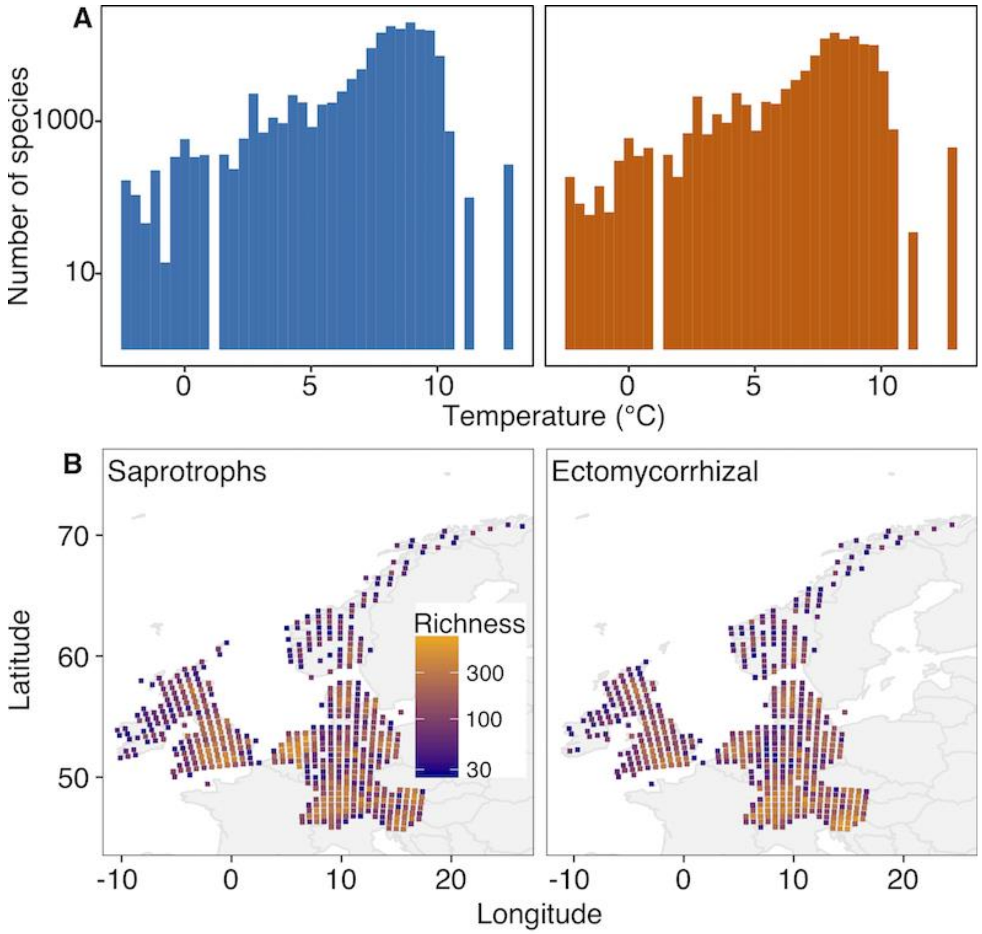

Supplementary Figure 4. Density histogram and map of the number of species. (**A**) Density histogram of the number of species along the mean annual temperature; saprotrophs in blue, ectomycorrhizal fungi in orange. Note log_{10}-transformation of the y-axis and richness. (**B**) Map with occupied grids after data preparation (see Method section) and number of species (richness). Lighter colors indicate higher number of species. Maps were created using the R package ggplot2 with the function *borders*. Source data are provided Supplementary Data 4.

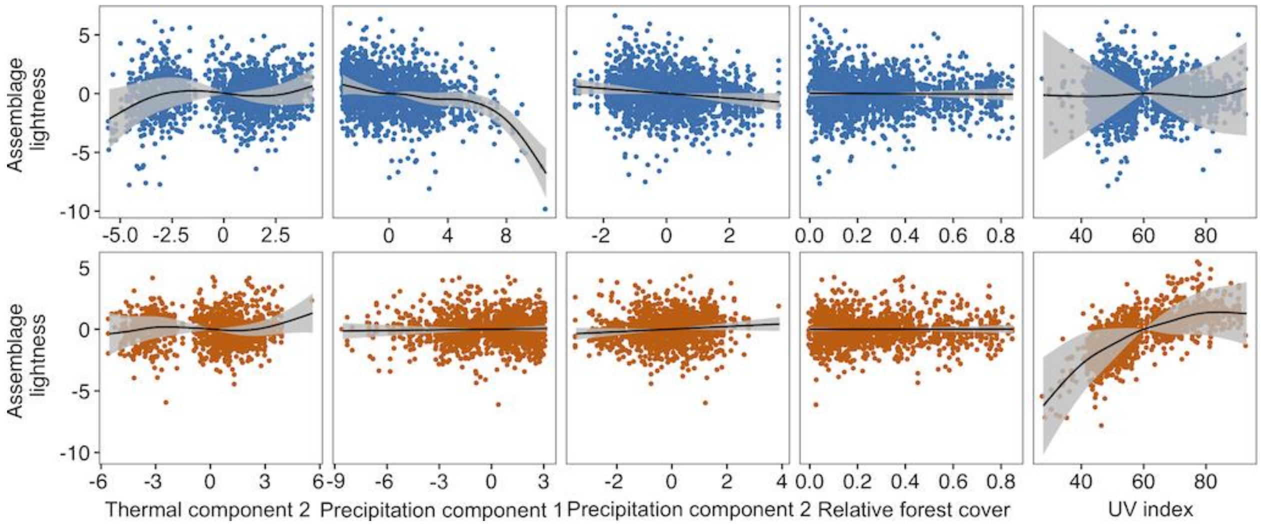

Supplementary Figure 5. Partial effect of covariates on mushroom color lightness. The top row displays effects for saprotrophs (blue), the bottom row for ectomycorrhizal fungi (orange). Plots are based on fits of the generalized additive models (GAM, Table 1). The gap within the thermal component 2 corresponds to the climatological difference between the British Isles and the European mainland, which differ in seasonality variation due to the degree of continentality. Source data are provided in Supplementary Data 4.

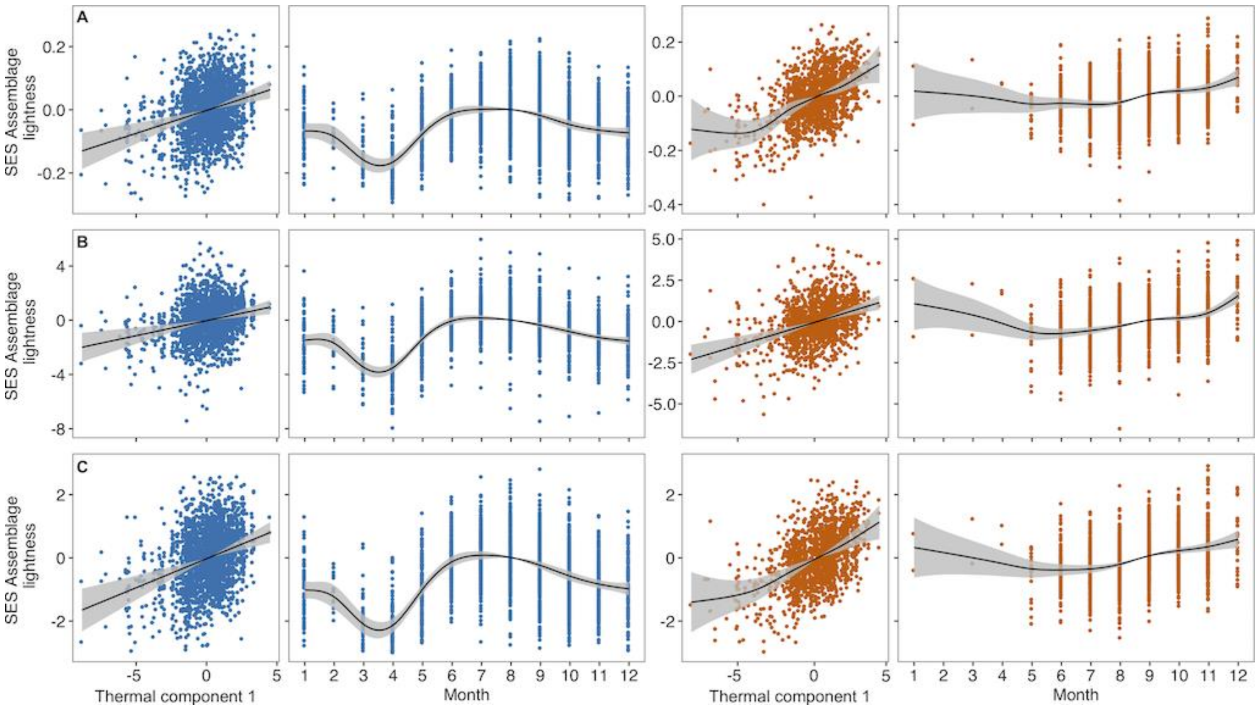

Supplementary Figure 6. Partial effects of temperature and seasonality on standardized effect sizes (SES). Standardized effect sizes of mushroom color lightness were calculated based on 100 randomizations of the community matrix (3,054 species) using the three null models: (**A**) independent swap (**B**), richness, and (**C**) frequency. Saprotroph responses are shown in the two columns on the left (blue); ectomycorrhizal responses in the two columns on the right (orange). For statistics see Table 1. Source data are provided in Supplementary Data 4.

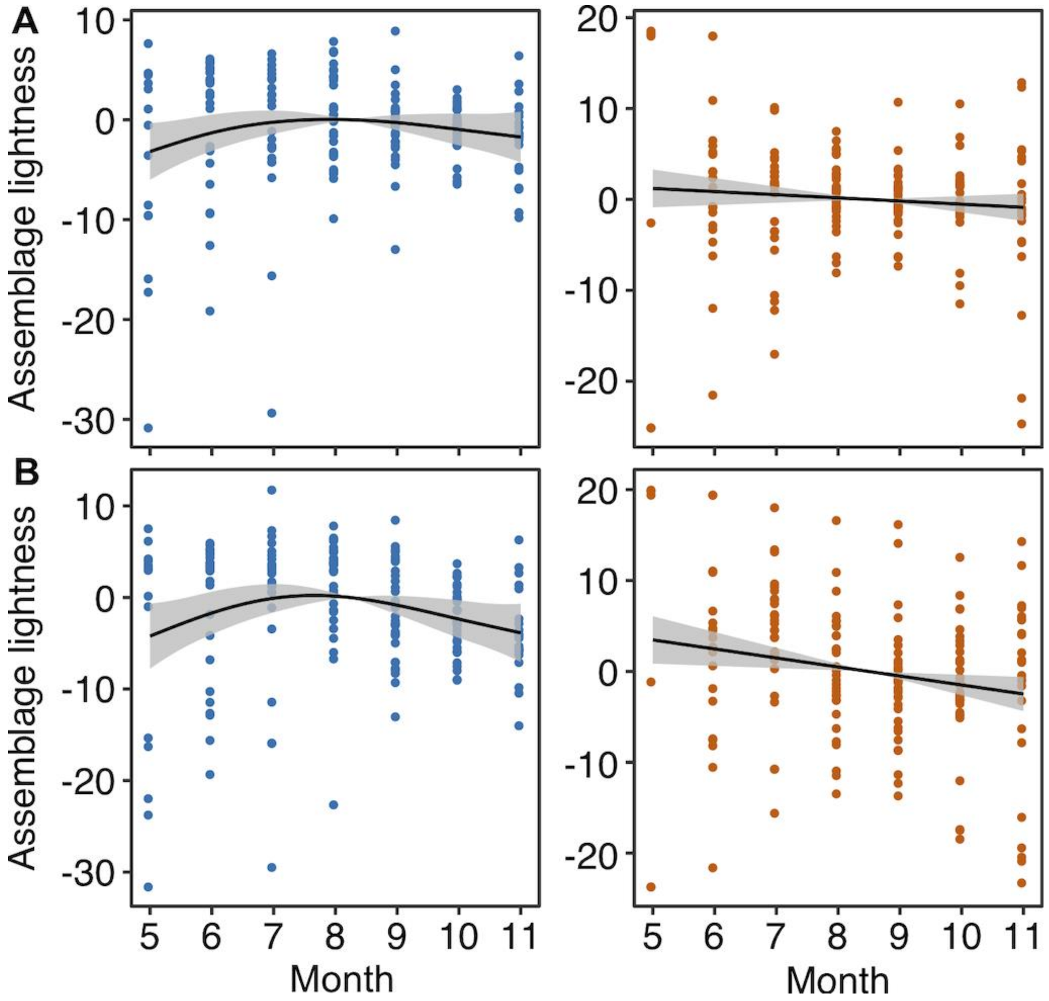

Supplementary Figure 7. Partial effects of seasonality on mushroom color lightness for a local dataset. Effect of months on the mushroom color lightness of saprotrophs (left, blue) and ectomycorrhizal fungi (right, orange). Mushroom color lightness was calculated based on (**A**) presence/absence data (generalized additive models: saprotroph: *F*-value=1.65, *p*-value=0.146; ectomycorrhizal: *F*-value=1.30, *p*-value=0.255), and (**B**) abundance data (generalized additive models: saprotroph: *F*-value=2.96, *p*-value=0.031; ectomycorrhizal: *F*-value=6.75, *p*-value=0.01, Supplementary Table 4). Slopes are estimates from generalized additive models (GAMs) with standard deviations. Source data are provided in Supplementary Data 6.

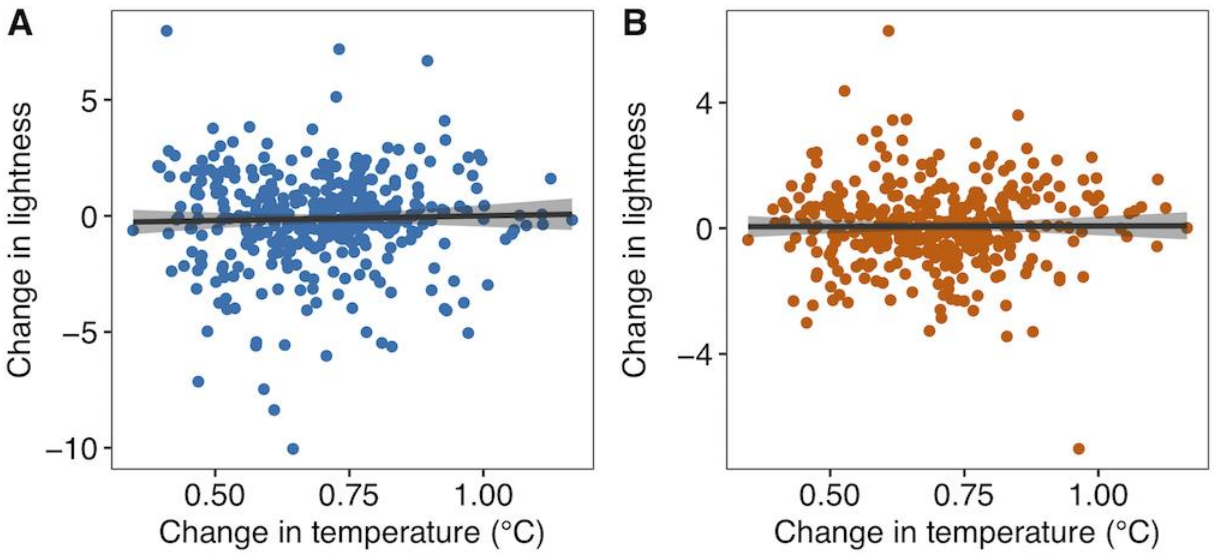

Supplementary Figure 8. Effects of climate warming on change in mushroom color lightness. To test for effects of climate warming on mushroom color lightness, we split our 40-year European dataset into two time intervals: 1970–1990 and 1991–2010. The number of species analyzed were as follows; ECM (1970-1990): 1167, ECM (1991-2010): 1371; SAP (1970-1990): 1497, SAP (1991-2010): 1621. The border between these two time intervals marks the approximate border of accelerated climate warming [5]. We calculated the change in temperature as the mean annual temperature of the first time-interval subtracted from the mean annual temperature of the second time-interval; the change in lightness is the mushroom color lightness of the first time-interval subtracted from the mushroom color lightness of the second time-interval. Effect of a change in temperature on a change in mushroom color lightness of (**A**) saprotrophic fungi (blue) and (**B**) ectomycorrhizal (orange) between the two time-intervals. Lines are linear model regressions with error bars displaying 95% confidence interval. Source data are provided in Supplementary Data 7.

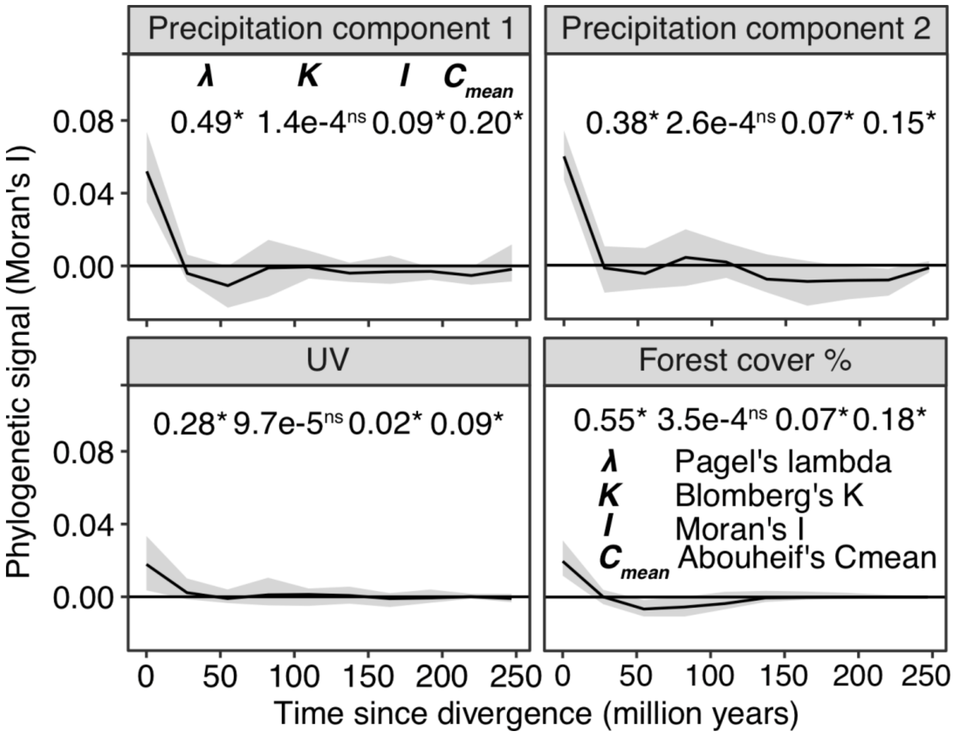

Supplementary Figure 9. Phylogenetic signal of species environmental variables (niche position). The phylogenetic signal was calculated as four different measures, and graphically displayed based on Moran's *I* (phylogenetic correlogram). Species which occupied at least 10 grid cells were used. Confidence interval (CI) based on 99 bootstraps. Significant phylogenetic signal was detectable only on very short phylogenetic distances, i.e., between closely related taxa (CI above null line). To estimate overall phylogenetic signal, we used (from left to right): Pagel's lambda (λ), Blomberg's K, Moran's I and Abouheif's C_{mean}. Source data are provided in Supplementary Data 1, 2 and 3.

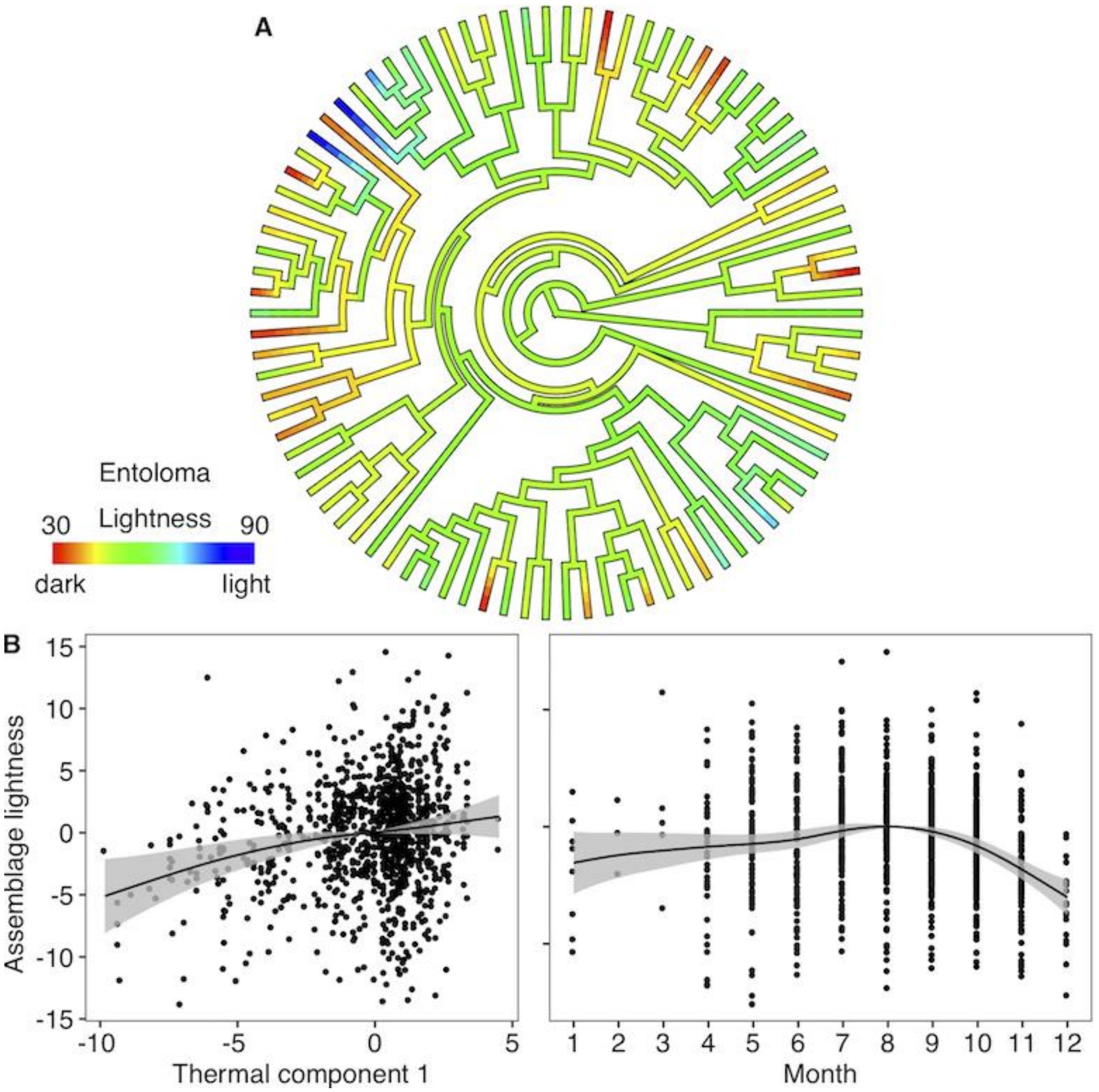

Supplementary Figure 10. Partial effects of temperature and seasonality on the widespread genus *Entoloma*. (**A**) Ancestral state estimation (function *contMap*, R package phytools) of the continuous lightness trait showing single-species or small clade divergences resulting in low phylogenetic signal (Fig. 5). (**B**) Partial effects of temperature (generalized additive models: *F*-value=4.26, *p*-value<0.007) and month (generalized additive model: *F*-value=15.43, *p*-value<0.001) on mushroom color lightness within the genus *Entoloma*, based on a generalized additive model (R^2=0.22). Model is based on 491 grids and 90 species. We additionally

considered space (geographical latitude and longitude) as a covariate (generalized additive model: F-value=3.19, p-value<0.001) and grid as random effect.

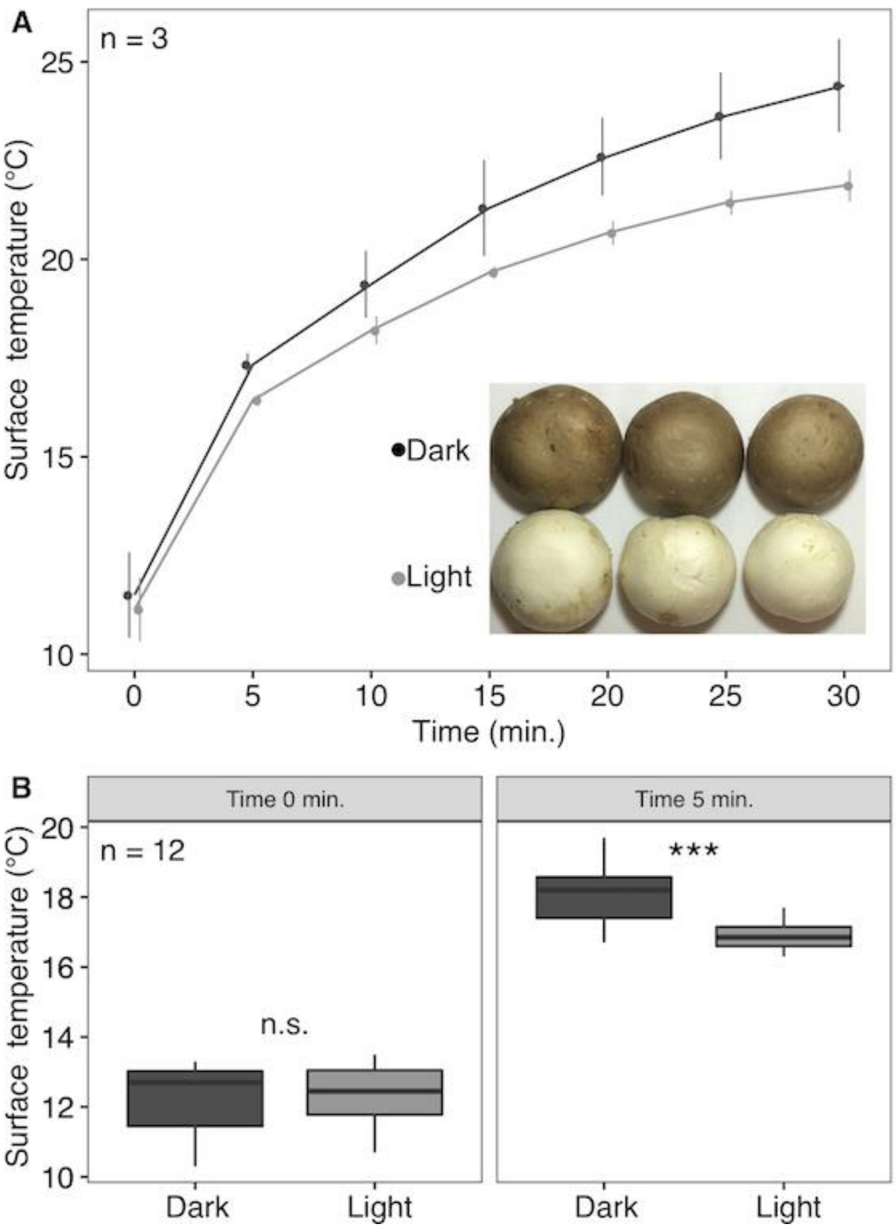

Supplementary Figure 11. Experimental effect of artificial radiation on mushroom cap surface temperature. (A) We first tested the temporal effect on mushroom cap surface temperature using 3 dark-colored and 3 light-colored caps of *Agaricus bisporus* individuals. We found that dark-colored mushrooms were warmer than light-colored mushrooms and that this difference increased over time. Error bars denote the upper and lower range of the standard deviation. (**B**) We then tested the warming difference based on 12 dark-and light-colored mushrooms and found significantly warmer mushroom caps of the dark variety after 5 minutes

(difference between means: 1.21 °C) (linear model: 0 min: *t*-value=0.214, *p*-value=0.833; 5 min: *t*-value=-4.23, *p*-value<0.001). Note finally that this result is consistent with studies demonstrating that dark-pigmented insects and yeasts heated up faster and reached higher temperatures than lighter individuals following irradiation [6]. Boxplots denote the median (horizontal line) and interquartile range (colored box); whiskers show three times the interquartile range. Source data are provided in Supplementary Data 8.

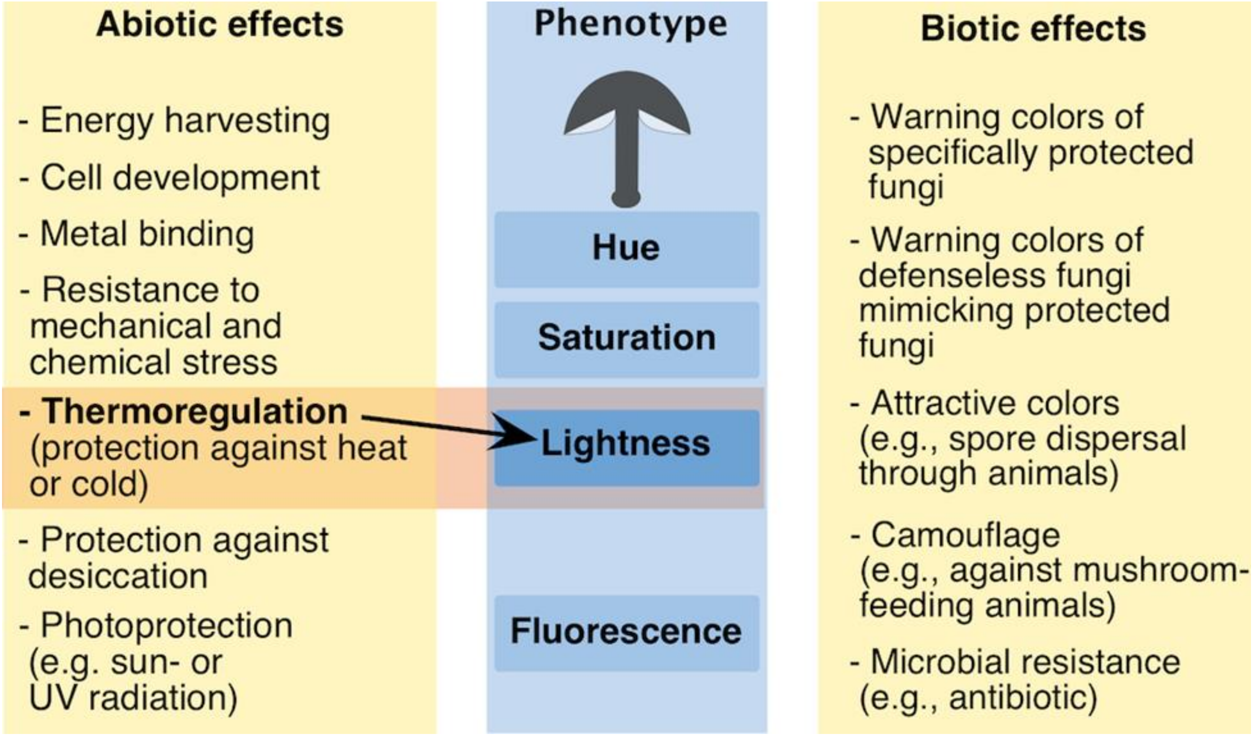

Supplementary Figure 12. Overview of possible abiotic and biotic effects on the mushroom color phenotype. Abiotic effects were adapted from Cordero *et al.* [7]; biotic effects from Caro [8]. The theory of "thermal melanism" is highlighted (see also Fig. 1).

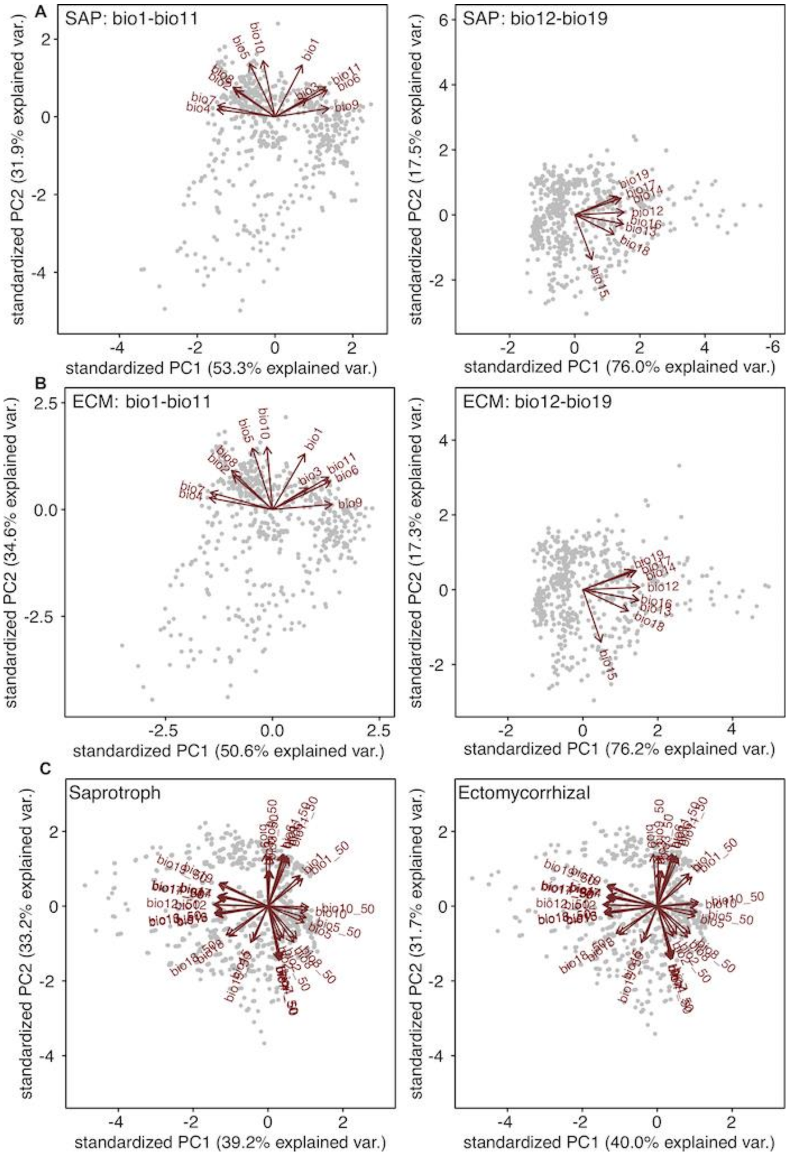

Supplementary Figure 13. Principal component analysis (PCA) for temperature and precipitation. PCAs were based on WorldClim variables for (**A**) saprotrophic (SAP) and (**B**) ectomycorrhizal fungi (ECM). The PCA for the temperature variables of SAP and ECM fungi displays overall mean values on axis 2 (e.g., bio1, mean annual temperature) and temperature variability on axis 1 (e.g., bio4, temperature seasonality). The PCA for the precipitation variables for SAP and ECM displays overall mean values on axis 1 (e.g., bio12, annual precipitation) and variability on axis 2 (e.g., bio 15, precipitation seasonality). (**C**) All WorldClim variables based

on 50 km × 50 km grids (denoted by the addition: '_50') and 10 km × 10 km subgrids occupied by fungi averaged based on 50 × 50 km grids. Displayed are standardized principal components with explained variances in brackets. Source data are provided in Supplementary Data 4.

Supplementary Table 1. Data overview. The total number of species in the European fungal dataset; the number of mushroom-forming species (mushroom = fruit body with cap and stipe, example: Fig. 2) with color data; and the number of mushroom-forming species with color and sequence data. Percent values refer to the "Mushroom-forming species" dataset. HSL = hue, saturation, and lightness.

	**Full dataset**	**Mushroom-forming species**	**Mushroom-forming species & HSL data**	**DNA sequences available**
Ectomycorrhizal fungi	1,989	1,770	1,401 (79%)	1,011 (57%)
Saprotrophic fungi	3,481	2,289	1,653 (72%)	1,046 (46%)
Other (parasitic, endophytic, unknown)	261	-	-	-
Total	5,831	4,059	**3,054 (75%)**	**2,057 (51%)**

Supplementary Table 2. Test for differences in color lightness between species of nutritional modes. Phylogenetic linear regressions based on different models of trait evolution based on the maximum likelihood phylogeny (2,057 species). BM, Brownian motion; OU, Ornstein-Uhlenbeck; Lambda, Pagel's lambda. We fitted the Lambda model with 100 alternative trees. Effects are presented as *t*-values (estimates divided by the respective standard error), significant effect sizes are highlighted in bold. Superscripts indicate rank of model selection based on AIC scores. SAP = Saprotroph; ECM = Ectomycorrhizal; ML = maximum likelihood. Source data are provided in Supplementary Data 1, 2 and 3.

	Lambda[1]						OUrandomRoot[2]		BM[3]	
	t-value			*p*-value			*t*-value	*p*-value	*t*-value	*p*-value
	ML	lower	upper	ML	lower	upper				
Intercept	**7.35**	**4.73**	**6.74**	**<0.001**	**<0.001**	**<0.001**	**132.55**	**<0.001**	0.13	0.894
SAP vs. ECM	**4.06**	**3.07**	**4.25**	**<0.001**	**<0.001**	**0.002**	**10.63**	**<0.001**	1.32	0.187

Supplementary Table 3. Test for differences in mushroom color lightness between nutritional modes. Linear mixed effects model to test for differences in assemblage-based mushroom color lightness between saprotrophic and ectomycorrhizal fungi. A random effect of grid cell was incorporated due to repeated temporal sampling of the nutritional modes within grid cells.

	t-value	*p*-value
Intercept	**822.04**	**<0.001**
Saprotroph vs Ectomycorrhizal	**126.39**	**<0.001**

Supplementary Table 4. Seasonality and mushroom color lightness on local scale. Here we use generalized additive models (GAMs) based on a standardized local scale data set (La Chanéaz). Effects of mushroom color lightness in response to month are given for saprotrophic and ectomycorrhizal fungi. Effects are presented as *F*-values (*F*) and significant effects are emboldened (*p*=*p*-value). The assemblage-based calculation was based on an abundance community matrix of mushroom-forming fungi (194 ectomycorrhizal and 121 saprotrophic species). Results (standardized effect sizes) based on three null models are shown.

		**Mushroom color lightness (non-linear)**		**Standardized effect sizes Null models**					
				**Independent swap**		**Richness**		**Frequency**	
		F	*p*	*F*	*p*	*F*	*p*	*F*	*p*
Saprotroph	Month	**2.97**	**0.031**	**2.94**	**0.033**	**2.84**	**0.037**	**3.31**	**0.018**
	Adj. R^2	0.08		0.07		0.07		0.07	
Ecto-mycorrhizal	Month	**6.75**	**0.010**	**6.56**	**<0.001**	**6.66**	**0.011**	**3.76**	**0.011**
	Adj. R^2	0.03		0.11		0.03		0.06	

Supplementary Table 5. Phylogenetic regression of the effect of thermal components on species lightness. Thermal components were averaged for each species occurring at least on 10 grid cells (793 ectomycorrhizal and 869 saprotrophic species). Besides testing the overall effect of the thermal components on species lightness, we furthermore tested the interaction of the species' nutritional mode (see 'Statistical analyses' in the Methods for details). Note that the thermal components are significantly positive correlated with species lightness and that there is no difference in the response of this relationship between the nutritional modes. BM, Brownian motion; OU, Ornstein-Uhlenbeck; Lambda, Pagel's lambda. We fitted the Lambda model based on 100 alternative trees. Effects are presented as *t*-values (estimates divided by the respective standard error), and significant effects are emboldened. Superscripts indicate rank of model selection based on AIC scores.

	**Lambda[1]**						**OUrandomRoot[2]**		**BM[3]**	
	***t*-value**			***p*-value**						
	ML	lower	upper	ML	lower	upper	*t*-value	*p*-value	*t*-value	*p*-value
Intercept	**7.28**	**6.43**	**9.03**	**<0.001**	**<0.001**	**<0.001**	32.31	<0.001	0.14	0.892
Thermal component 1 (TC 1)	**2.60**	**2.25**	**2.58**	**0.009**	**0.010**	**0.025**	4.83	<0.001	4.01	<0.001
Thermal component 2 (TC 2)	**2.16**	**1.98**	**2.49**	**0.031**	**0.013**	**0.048**	1.74	0.081	4.21	<0.001
Nutritional mode: TC 1	-0.42	-0.46	-0.13	0.672	0.648	0.899	-0.30	0.767	4.23	<0.001
Nutritional mode: TC 2	0.85	0.81	1.18	0.397	0.236	0.420	1.87	0.061	0.50	0.619

Supplementary Table 6. Linear mixed effects model to test of differences in species lightness between saprotroph and ectomycorrhizal fungi and the effect of the thermal components. Phylogenetic regressions might provide incorrect degrees of freedom if clades share the same trait owing to common descent. According to Grafen [9], each radiation (e.g., radiation of ectomycorrhizal fungal clade) should be treated as an independent data point with one degree of freedom (‘radiation principle’). Thus, in addition to the phylogenetic regressions (Supplementary Table 5), we also used a linear mixed model (LMM) that included “genus” clades as a random effect in the function *lmer* from the R package lmerTest (“+ (1|genus)”). We used the genus because we found increased phylogenetic signal mainly within genera (Fig. 5). (**A**) LMM to test for lightness differences between nutritional modes. (**B**) LMM to test for the effects of the thermal components (niche positions) on mushroom lightness. Degrees of freedom (DF) were calculated based on type III analysis of variance table with Satterthwaite's method (typically applied in mixed models [10]; here using function *anova*).

	DF	*t*-value	*p*-value
**A)**			
Intercept	92	**36.10**	**<0.001**
Nutritional mode – SAP vs. ECM		**3.75**	**<0.001**
**B)**			
Intercept	1049	**32.38**	**<0.001**
Thermal component 1 (TC 1)	1659	**1.98**	**0.048**
Thermal component 2 (TC 2)	1636	**2.00**	**0.045**
Nutritional mode: TC 1	1653	-0.45	0.652
Nutritional mode: TC 2	1642	0.81	0.417

Supplementary Table 7. Pairwise correlation coefficients (*r*) among environmental variables. Upper triangle (orange) for ectomycorrhizal fungi and lower triangle (blue) for saprotrophic fungi. (**A**) Correlation coefficients for the assemblage-based approach. Source data are provided in Supplementary Data 4. (**B**) Correlation coefficients for the cross-species approach (see 'Statistical analyses' in the Methods for details). Source data are provided in Supplementary Data 1, 2, and 3. Correlation coefficients above 0.6 or below –0.6 are emboldened.

	TC 1	TC 2	PC 1	PC 2	UV	RFC	Month
A)							
Thermal component 1 (TC 1)	-	0.22	–0.39	0.14	0.25	0.05	–0.01
Thermal component 2 (TC 2)	0.18	-	–0.04	**0.66**	0.55	0.50	–0.12
Precipitation component 1 (PC 1)	–0.40	–0.06	-	–0.05	0.43	-0.002	–0.04
Precipitation component 2 (PC 2)	0.10	**0.69**	–0.06	-	0.31	0.24	–0.06
UV	0.15	0.58	0.45	0.36	-	0.28	–0.10
Relative forest cover (RFC)	0.02	0.52	–0.02	0.29	0.30	-	–0.08
Month	–0.07	–0.07	0.05	–0.01	–0.10	–0.05	-
B)							
Thermal component 1 (TC 1)	-	0.25	**–0.62**	**–0.78**	0.06	–0.17	
Thermal component 2 (TC 2)	0.05	-	0.08	0.10	**0.73**	**0.62**	
Precipitation component 1 (PC 1)	**–0.63**	–0.02	-	0.51	0.52	0.48	
Precipitation component 2 (PC 2)	**–0.74**	0.27	0.36	-	0.04	0.23	
UV	0.02	**0.78**	0.38	0.10	-	**0.75**	
Relative forest cover (RFC)	–0.46	0.56	0.46	0.41	**0.60**	-	

Supplementary References

1. Ludwig, E. *Pilzkompendium, Bd. 1 Abbildungen: Die kleineren Gattungen der Makromyzeten mit lamelligem Hymenophor aus den Ordnungen Agaricales, Boletales und Polyporales*. (Eching, 2000).
2. Ludwig, E. *Pilzkompendium. Band 2. Die größeren Gattungen der Agaricales mit farbigem Sporenpulver (ausgenommen Cortinariaceae). Beschreibungen+ Abbildungen. Fungicon-Verlag* (2007).
3. Ludwig, E. *Pilzkompendium. Band 3. Beschreibungen. Die übrigen Gattungen der Agaricales mit weißem Sporenpulver.* (Berlin, Fungicon Verlag, 2012).
4. Ludwig, E. *Pilzkompendium. Band 4. Cortinariaceae I - Cortinarius (part I), Galerina, Hebeloma, Hebelomina, Inocybe, Phaeogalera*. (Berlin, Fungicon Verlag, 2017).
5. Mann, M. E., Bradley, R. S. & Hughes, M. K. Global-scale temperature patterns and climate forcing over the past six centuries. *Nature* **392,** 105 (2004).
6. Willmer, P. G. & Unwin, D. M. Field analyses of insect heat budgets: Reflectance, size and heating rates. *Oecologia* **50,** 250–255 (1981).
7. Cordero, R. J. B. & Casadevall, A. Functions of fungal melanin beyond virulence. *Fungal Biol. Rev.* **31,** 99–112 (2017).
8. Caro, T. Wallace on Coloration: Contemporary Perspective and Unresolved Insights. *Trends Ecol. Evol.* **32,** 23–30 (2017).
9. Grafen, A. The phylogenetic regression. *Philos. Trans. R. Soc. Lond. B. Biol. Sci.* **326,** 119–157 (1989).
10. Keselman, H. J., Kowalchuk, R. K., Algina, J. & Wolfinger, R. D. The analysis of repeated measurements: A comparison of mixed-model Satterthwaite F tests and a nonpooled adjusted degrees of freedom multivariate test. Commun. Stat. - Theory Methods 28, 2967–2999 (1999).
