# Supplementary material for: European mushroom assemblages are darker in cold climates
Source: Nat Commun. 2019 Jun 28;10:2890. doi: 10.1038/s41467-019-10767-z (PMC6599080; doi:10.1038/s41467-019-10767-z)
Supplement: Supplementary file 2 — Reporting Summary [file 41467_2019_10767_MOESM2_ESM.pdf]

nature research

Corresponding author(s): Claus Bässler and Franz-S. Krah

Last updated by author(s): 23.05.2019

Reporting Summary

Nature Research wishes to improve the reproducibility of the work that we publish. This form provides structure for consistency and transparency in reporting. For further information on Nature Research policies, see Authors & Referees and the Editorial Policy Checklist .

Statistics

For all statistical analyses, confirm that the following items are present in the figure legend, table legend, main text, or Methods section.

n/a	Confirmed	
☐	☒	The exact sample size (n) for each experimental group/condition, given as a discrete number and unit of measurement
☐	☒	A statement on whether measurements were taken from distinct samples or whether the same sample was measured repeatedly
☐	☒	The statistical test(s) used AND whether they are one- or two-sided<br>*Only common tests should be described solely by name; describe more complex techniques in the Methods section.*
☐	☒	A description of all covariates tested
☐	☒	A description of any assumptions or corrections, such as tests of normality and adjustment for multiple comparisons
☐	☒	A full description of the statistical parameters including central tendency (e.g. means) or other basic estimates (e.g. regression coefficient) AND variation (e.g. standard deviation) or associated estimates of uncertainty (e.g. confidence intervals)
☐	☒	For null hypothesis testing, the test statistic (e.g. F, t, r) with confidence intervals, effect sizes, degrees of freedom and P value noted<br>*Give P values as exact values whenever suitable.*
☒	☐	For Bayesian analysis, information on the choice of priors and Markov chain Monte Carlo settings
☒	☐	For hierarchical and complex designs, identification of the appropriate level for tests and full reporting of outcomes
☐	☒	Estimates of effect sizes (e.g. Cohen's d, Pearson's r), indicating how they were calculated

Our web collection on statistics for biologists contains articles on many of the points above.

Software and code

Policy information about availability of computer code

Data collection	Pipette (Stefan Trost Media, http://www.sttmedia.com/)
Data analysis	R version 3.5.1

For manuscripts utilizing custom algorithms or software that are central to the research but not yet described in published literature, software must be made available to editors/reviewers. We strongly encourage code deposition in a community repository (e.g. GitHub). See the Nature Research guidelines for submitting code & software for further information.

Data

Policy information about availability of data

All manuscripts must include a data availability statement. This statement should provide the following information, where applicable:

- Accession codes, unique identifiers, or web links for publicly available datasets
- A list of figures that have associated raw data
- A description of any restrictions on data availability

Provide your data availability statement here.

Field-specific reporting

Please select the one below that is the best fit for your research. If you are not sure, read the appropriate sections before making your selection.

☐ Life sciences ☐ Behavioural & social sciences ☒ Ecological, evolutionary & environmental sciences

For a reference copy of the document with all sections, see nature.com/documents/nr-reporting-summary-flat.pdf

Ecological, evolutionary & environmental sciences study design

All studies must disclose on these points even when the disclosure is negative.

Study description	Test whether the lightness of mushrooms is related to climate using a dataset of 3.2 million observations in 3,054 species with 29,490 color samples from 50 km x 50 km grids across Europe.
Research sample	We used an existing dataset of fungal occurrences of Europe (Andrew et al. 2017, Fungal Biology Reviews). Research sample are mushroom-forming fungi and their occurrences based on herbarium, museum and citizen-science data.
Sampling strategy	Over 7.3 million national-scale fruit body records from nine European countries were compiled from multiple sources including museums, educational institutions, mycological societies, and citizen-science projects. For further details see (Andrew et al. 2017, Fungal Biology Reviews).<br>We sampled 9 color values for each mushroom cap to retrieve a robust average color value. We demonstrate that these average values discriminate lightness variability between species.
Data collection	We additionally sampled color values. The data was collected mainly by the Franz-S. Krah and Antje Heideroth. Relevant websites with colored mushroom images were searched. Mushroom images were selected based on quality checks. Using color picker software (pipette) at least 9 color samples were taken for each mushroom cap. The RGB values were transformed into HSL values and an average hue, saturation and lightness value was calculated for each species. Lightness was used for further analysis.
Timing and spatial scale	1970 - 2010<br>Continental scale, including 8 European countries
Data exclusions	We excluded the following data:<br>1) Community analysis:<br>a) Species where we could not find high-quality images for color sampling<br>1,770 => 1,401 for ectomycorrhizal fungi;<br>2,289 => 1,653 for saprotrophic fungi<br>b) Grids with less than 25 species (743 grids => 549 and 522 saprotrophic and ectomycorrhizal fungi, respectively)<br>2) Climate change analysis: reduced to grids with data for both time intervals (743 grids => 356)<br>3) Phylogenetic analysis: Species where no genome sequences were available:<br>1,401 => 1,011 for ectomycorrhizal<br>1,653 => 1,046 for saprotrophic fungi<br>4) We did not include Estonia because of low overall data density (Andrew et al. 2017, Fungal Biology Reviews)
Reproducibility	Mushroom heating experiment: We repeated the experiment using 3 or 12 mushroom replicates.
Randomization	We used three null model approaches: "frequency", "richness" and "independentswap" null models.
Blinding	No blinding was used because the color of the mushrooms is always visible.

Did the study involve field work? ☐ Yes ☒ No

Reporting for specific materials, systems and methods

We require information from authors about some types of materials, experimental systems and methods used in many studies. Here, indicate whether each material, system or method listed is relevant to your study. If you are not sure if a list item applies to your research, read the appropriate section before selecting a response.

Materials & experimental systems

n/a	Involved in the study
☒	☐ Antibodies
☒	☐ Eukaryotic cell lines
☒	☐ Palaeontology
☒	☐ Animals and other organisms
☒	☐ Human research participants
☒	☐ Clinical data

Methods

n/a	Involved in the study
☒	☐ ChIP-seq
☒	☐ Flow cytometry
☒	☐ MRI-based neuroimaging
