# Supplementary material for: European mushroom assemblages are darker in cold climates
Source: Nat Commun. 2019 Jun 28;10:2890. doi: 10.1038/s41467-019-10767-z (PMC6599080; doi:10.1038/s41467-019-10767-z)
Supplement: Supplementary file 3 — Description of Additional Supplementary Files [file 41467_2019_10767_MOESM3_ESM.pdf]

Description of Additional Supplementary Files

File Name: Supplementary Data 1
Description: Species color data

File Name: Supplementary Data 2
Description: Phylogenetic tree

File Name: Supplementary Data 3
Description: Alternative phylogenetic hypotheses

File Name: Supplementary Data 4
Description: European species data set

File Name: Supplementary Data 5
Description: Color reliability test data

File Name: Supplementary Data 6
Description: Local species data set

File Name: Supplementary Data 7
Description: European species data set splitted for time shift analysis

File Name: Supplementary Data 8
Description: Experimental heating data set
